# Positive Allosteric Modulation of Kv Channels by Sevoflurane: *Insights into the Structural Basis of Inhaled Anesthetic Action*

**DOI:** 10.1371/journal.pone.0143363

**Published:** 2015-11-24

**Authors:** Qiansheng Liang, Warren D. Anderson, Shelly T. Jones, Caio S. Souza, Juliana M. Hosoume, Werner Treptow, Manuel Covarrubias

**Affiliations:** 1 Department of Neuroscience, Sidney Kimmel Medical College of Thomas Jefferson University, Philadelphia, Pennsylvania, United States of America; 2 Farber Institute for Neuroscience, Sidney Kimmel Medical College of Thomas Jefferson University, Philadelphia, Pennsylvania, United States of America; 3 Laboratório de Biologia Teórica e Computacional, Departamento de Biologia Celular, Universidade de Brasília, Brasília, Brasil; University at Buffalo, UNITED STATES

## Abstract

Inhalational general anesthesia results from the poorly understood interactions of haloethers with multiple protein targets, which prominently includes ion channels in the nervous system. Previously, we reported that the commonly used inhaled anesthetic sevoflurane potentiates the activity of voltage-gated K^+^ (Kv) channels, specifically, several mammalian Kv1 channels and the *Drosophila* K-Shaw2 channel. Also, previous work suggested that the S4-S5 linker of K-Shaw2 plays a role in the inhibition of this Kv channel by *n*-alcohols and inhaled anesthetics. Here, we hypothesized that the S4-S5 linker is also a determinant of the potentiation of Kv1.2 and K-Shaw2 by sevoflurane. Following functional expression of these Kv channels in *Xenopus* oocytes, we found that converse mutations in Kv1.2 (G329T) and K-Shaw2 (T330G) dramatically enhance and inhibit the potentiation of the corresponding conductances by sevoflurane, respectively. Additionally, Kv1.2-G329T impairs voltage-dependent gating, which suggests that Kv1.2 modulation by sevoflurane is tied to gating in a state-dependent manner. Toward creating a minimal Kv1.2 structural model displaying the putative sevoflurane binding sites, we also found that the positive modulations of Kv1.2 and Kv1.2-G329T by sevoflurane and other general anesthetics are T1-independent. In contrast, the positive sevoflurane modulation of K-Shaw2 is T1-dependent. *In silico* docking and molecular dynamics-based free-energy calculations suggest that sevoflurane occupies distinct sites near the S4-S5 linker, the pore domain and around the external selectivity filter. We conclude that the positive allosteric modulation of the Kv channels by sevoflurane involves separable processes and multiple sites within regions intimately involved in channel gating.

## Introduction

Every year, millions of patients around the world undergo general anesthesia to perform major surgeries. However, the mechanisms of general anesthesia, which involve multiple targets, are poorly understood. Also, although general anesthesia is considered generally safe, the drugs currently used lack specificity and have narrow therapeutic indices. Thus, it is necessary to understand anesthesia at all levels to help develop specific, effective and less toxic general anesthetics. Diverse ion channels in the nervous system are likely to be major targets of general anesthetics [1]. Among them, neurotransmitter-gated ion channels and non-gated K^+^ channels have received most of the attention [1, 2]; however, recent work continues to suggest that voltage-gated ion channels are also important players in general anesthesia [3–7].

In particular, recent discoveries have reassessed the likely role of certain Kv1 channels in general anesthesia [7–9]. Kv1.2 channels encoded by *Kcna2* gene are widely expressed in the brain and mainly localize in the axon initial segment, the juxtaparanodal region of myelinated axons and axon terminals [10–12]. In these locations, heteromultimeric Kv channels including Kv1.1 and Kv1.2 subunits help regulate action potential threshold, repolarization, propagation and firing patterns [12]. *Kcna2*-null mice exhibit a severe brainstem seizure phenotype, which is often lethal [12]. The findings of Alkire et al. [8] and Lioudyno et al. [9] are especially significant. Whereas the first study found that infusion of an anti-Kv1.2 antibody into the CMT of the rat reverses anesthesia, the second reported a similar result upon infusion of a specific neurotoxin to block Kv1.1, Kv1.3 and Kv1.6 channels. Moreover, tonic firing of action potentials in neurons of the CMT is inhibited by sevoflurane, which lengthens the interspike interval, and this inhibition is prevented by the aforementioned Kv1-specific neurotoxin.

Sevoflurane is commonly used in human general anesthesia and is a unique positive modulator of several Kv1 channels and the *Drosophila* K-Shaw2 channel in heterologous expression systems [7, 9]. At relevant concentrations, it induces a negative shift in the conductance-voltage relation and increases the maximum conductance. By contrast, most other common general anesthetics either interact weakly or inhibit Kv channels (namely, K-Shaw2). Since K^+^ channel activity opposes excitation, the unique potentiation of Kv channels by sevoflurane could contribute to anesthesia. Here, we investigated the structural basis of this positive modulation in the mammalian Kv1.2 channel. Previous work strongly implicated the S4-S5 linker as a determinant of the inhibition of K-Shaw2 by general anesthetics [6, 13]. The S4-S5 linker connects the voltage-sensing domain to the pore domain of the Kv channels and is responsible for the electromechanical coupling that controls voltage-dependent gating [14, 15]. In this study, we investigated the contributions of the S4-S5 linker to the positive modulation of the Kv1.2 and K-Shaw2 channels by sevoflurane. The results revealed that discrete structural changes in this linker drastically affect the response of these Kv channels to the anesthetic by altering electromechanical coupling. Other results strongly suggest additional novel sites, which are necessary to fully explain the modulation of Kv channels by sevoflurane. Supporting a multisite model of general anesthetic action, molecular dynamics, docking simulations and free energy estimations demonstrate two significant classes of sevoflurane binding sites. One set in the pore domain (including the C-terminal side of the S4-S5 linker) and another near the external selectivity filter of the Kv1.2 channel. We propose that positive multisite allosteric modulation of Kv channel gating by sevoflurane plays a significant role in general anesthesia.

## Materials and Methods

### Molecular biology and heterologous expression

Plasmid maintenance, mutagenesis, sequence, RNA synthesis and oocyte microinjection were carried out as previously described [6, 7].

### Ethics Statement

*Xenopus laevis* surgeries were performed according to a protocol approved by the Thomas Jefferson University IACUC.

### Electrophysiology

Whole-oocyte currents were recorded at room temperature (21–23°C) under two-electrode voltage-clamp conditions (OC-725C, Warner Instrument, Hamden, CT) according to established procedures [6, 7]. Data acquisition, leak subtraction and initial analysis were performed using pClamp 9.2 and 10.3 (Molecular Devices, Sunnyvale, CA). The pipette was filled with 3 M KCl. The extracellular solution was ND96 (in mM): 96 NaCl, 2 KCl, 1.8 CaCl_2_, 1 MgCl_2_, 5 HEPES, 2.5 sodium pyruvate, adjusted to pH 7.4 with NaOH. ND96 and *n*-butanol were delivered using a gravity-driven perfusion system. Volatile anesthetics were delivered manually using a Hamilton gastight syringe (Hamilton, Reno, NV). The preparation and dilution of all drugs were done as previously described [6, 7].

### Data analysis

Data analysis, plotting and curve-fitting were performed in Origin 9.1 (OriginLab, Northampton, MA).

*G-V relations*. Peak chord conductance (*G* = *I/*[*V*_c_−*V*_r_]) was determined assuming -95 mV as the reversal potentials (*V*_r_). *I* is the peak current and *V*_c_ is the command voltage. *G*-*V* relations were then described by assuming this form of the Boltzmann equation:

$$G=\frac{G_{max}}{\begin{array}{c} 1+e^{\frac{\left(V_{c}-V_{1/2}\right)}{k}} \end{array}}$$

or the double Boltzmann equation:

$$G=\frac{G_{max1}}{1+e^{\frac{V_{c}-V_{1/2,1}}{k_{1}}}}+\frac{G_{max2}}{1+e^{\frac{V_{c}-V_{1/2,2}}{k_{2}}}}$$

where *G*_max_ is the maximum conductance; *V*_1/2_ is the voltage at which the conductance is at 50% of its maximal amplitude; and *k* is the slope factor. The following equation was used to calculate the equivalent gating charge: *z* = *RT*/*Fk* = 25.5/*k*, where *R*, *T* and *F* are the gas constant, absolute temperature and Faraday constant, respectively. The *V*_med_ was also determined to evaluate multiphasic *G*-*V* relations in a curve fitting-independent manner. *V*_med_ is the voltage at which the total conductance (*G*_max1_ + *G*_max2_) is at its midpoint (median). This determination allowed semiquantitative assessment of multiphasic *G*-*V* relation shifts without the ambiguity typically associated with curve-fitting involving a large number of correlated adjustable parameters.

All *G*-*V* data were obtained from paired sets (same oocyte in the absence and presence of sevoflurane). Measurement of the control *G*-*V* relation was followed by determination of a stable response to sevoflurane (current evoked by a depolarizing step to +60 mV, before and after exposure to sevoflurane) and subsequent measurement of the experimental *G*-*V* relation. All parameters extracted from the analysis of these relations were compared in a pairwise manner.

**Fig 1:**
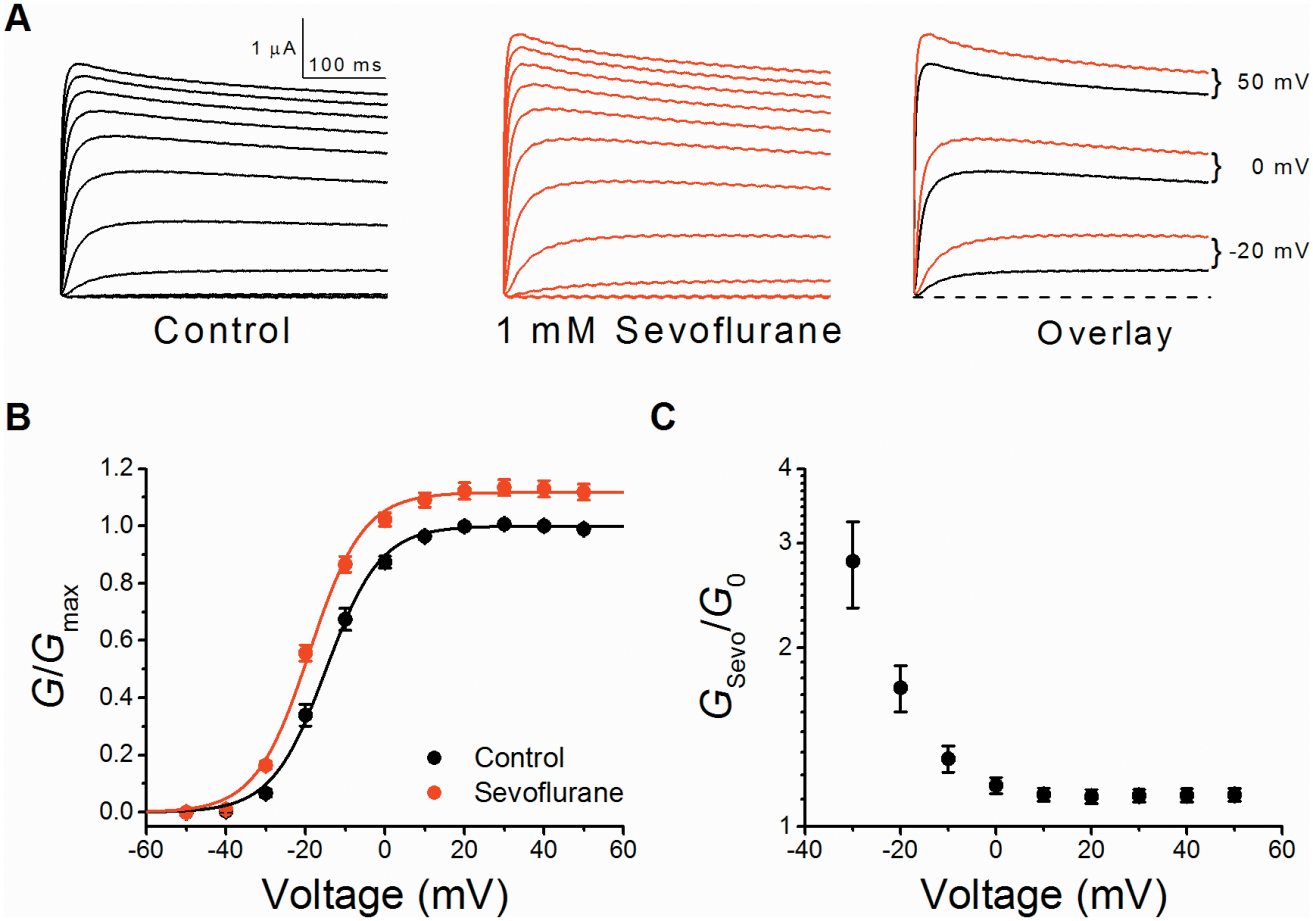
Positive modulation of the Kv1.2 conductance by sevoflurane. (A) Families of whole-oocyte Kv1.2 currents before (*left*) and after exposure to 1 mM sevoflurane (*center*). Currents were evoked by step depolarizations from a holding voltage of -100 mV. The steps were delivered in increments of 10 mV from -50 to +50 mV. The overlay (*right*) directly compares selected currents in the absence (*black*) and presence (*red*) of sevoflurane (currents evoked by steps to the indicated voltages). (B) Normalized *G*-*V* relations of Kv1.2 in the absence (*black*) and presence of 1 mM sevoflurane (*red*) (N = 6). The solid lines are the best-fit Boltzmann functions. *G*_max_ is the control maximum conductance before exposure to sevoflurane (Materials and Methods). The mean best-fit parameters are summarized in Table A in S1 File. (C) The voltage dependence of the Kv1.2 conductance ratio (*G*_Sevo_/*G*_0_). This ratio was calculated from paired measurements of the *G*-*V* relations before (*G*_0_) and after exposure to sevolfurane (*G*_Sevo_).

To display the results from different oocytes and estimate the magnitude of relative changes (before and after exposure to sevoflurane), individual *G*-*V* relations were normalized to the control *G*_max_ before exposure to sevoflurane (*G*/*G*_max_). Accordingly, a relative *G*/*G*_max_ = 1, <1 and >1 indicates no change, inhibition and potentiation, respectively (Table A in S1 File). Conductance ratio (*G*_Sevo_/*G*_0_, conductance in the presence of sevoflurane over control conductance) vs. voltage plots were additionally generated to directly visualize the *G*-*V* relation change (e.g., Fig 1C). All results were reported as mean ± SEM, and the paired Student *t*-test was used to evaluate the statistical significance of apparent differences, unless stated otherwise.

*Concentration-response relations*. Normalized dose-inhibition relations were described by assuming this form of the Hill equation:

$$\frac{I}{I_{0}}={\text{A}}_{1}+\frac{x^{n\text{H}}}{K^{n\text{H}}+x^{n\text{H}}}$$

or a double Hill equation:

$$\frac{I}{I_{0}}=1+\frac{1+{\text{A}}_{1}}{1+\;{\left(\frac{K_{1}}{x}\right)}^{n_{\text{H}1}}}+\frac{1+{\text{A}}_{2}}{1+\;{\left(\frac{K_{2}}{x}\right)}^{n_{\text{H}2}}}$$

where, *I/I*_0_ is the normalized current and *x* is the drug concentration, *K* (apparent dissociation equilibrium constant) is the drug concentration that induces 50% inhibition/potentiation, and *n*_H_ is the index of cooperativity or Hill coefficient (Table 1). We do not report SEM for the estimated best-fit Hill equation parameters because complete concentration-response relations and the corresponding best-fit function cannot be constructed from an individual oocyte. Although the action of all anesthetics tested is generally reversible [7], it is often difficult to obtain complete washout of inhaled anesthetics when testing multiple concentrations on the same oocyte. This complication would produce a cumulative effect, which distorts the concentration-response relation. To avoid this problem, each data point on the concentration-response relation represents the mean ± SEM from several independent determinations on different oocytes at the indicated concentrations. For each anesthetic, therefore, there is only one best-fit estimate that collectively describes the combined results from several oocytes individually tested at different concentrations. This approach has produced reproducible results as reported previously [6, 7].

**Table 1:** Concentration-response parameters of the modulation of selected Kv channels by sevoflurane and other general anesthetics.

	*K*_1_ (mM)	*n*_H1_	A_1_	*K*_2_ (mM)	*n*_H2_	A_2_
**Kv1.2**						
Sevofurane	0.36	3.3	1.13			
*n*-butanol	78.4	1.3				
**Kv1.2 FRAKT**						
Sevofurane	0.33	2.0	1	1.45	4.0	2.50
Isoflurane	0.23	2.2	1	0.47	9.4	1.24
Halothane	0.16	1.6	1	0.63	7.9	1.24
Propofol	0.02	2.2	2.89			
*n*-butanol	100.5	1.5				
ΔT1-**Kv1.2 FRAKT**						
Sevofurane	0.32	2.9	1	1.84	3.8	3.17
Isoflurane	0.22	1.2	1	0.40	3.4	1.96
Halothane	0.23	1.0	1	0.77	3.4	0.93
Propofol	0.02	1.7	2.66			
*n*-butanol	91.9	1.6				
**Kv1.2 G329T**						
Sevofurane	0.40	1.6	1	2.2	2.8	2.20
**K-Shaw2** *						
Sevofurane	0.06	1	0.59	4	1	1.23
Isoflurane	1.69	2.1				
Halothane	0.26	1.3				
Chloroform	0.70	1.4				
Propofol	0.06	1.6				
*n*-butanol	10.1	1.8				
**ΔT1-K-Shaw2**						
Halothane	0.38	1.4				
*n*-butanol	16.4	1.5				

* Sevoflurane tested at -20 mV, from Barber et al. [7].

### Computational Methods

Kv1.2 models in the closed/resting and open/activated states were obtained from Delemotte et al. [16] and Treptow & Tarek [17]. Each model was previously acquired via molecular dynamics (MD) simulations of the published x-ray crystal structure [18]. Details and validation of the models are as reported in the original papers mentioned above. Structural models of the Kv1.2-G329T mutant were built on the basis of the mentioned Kv1.2 constructs using the *psfgen* package implemented in VMD [19]. After insertion in a lipid bilayer, each of the structures was equilibrated by MD runs using the program NAMD 2.9 [20] and applying CHARMM 36 force field [21]. Specifically, sevoflurane molecules were then docked against the generated ensemble of membrane-equilibrated channel structures in order to explore putative binding sites using *AutoDock Vina* [22]. The Linear Interaction Energy (LIE) method [23] was adopted for calculation of the binding free energies and, thus, the corresponding binding constants. Assuming that the predicted binding sites act independently, these binding constants were computed for individual sites in different functional conformations. Additional details are provided under S1 File.

## Results

### Screening the effects of general anesthetics on the Kv1.2 current

**Fig 2:**
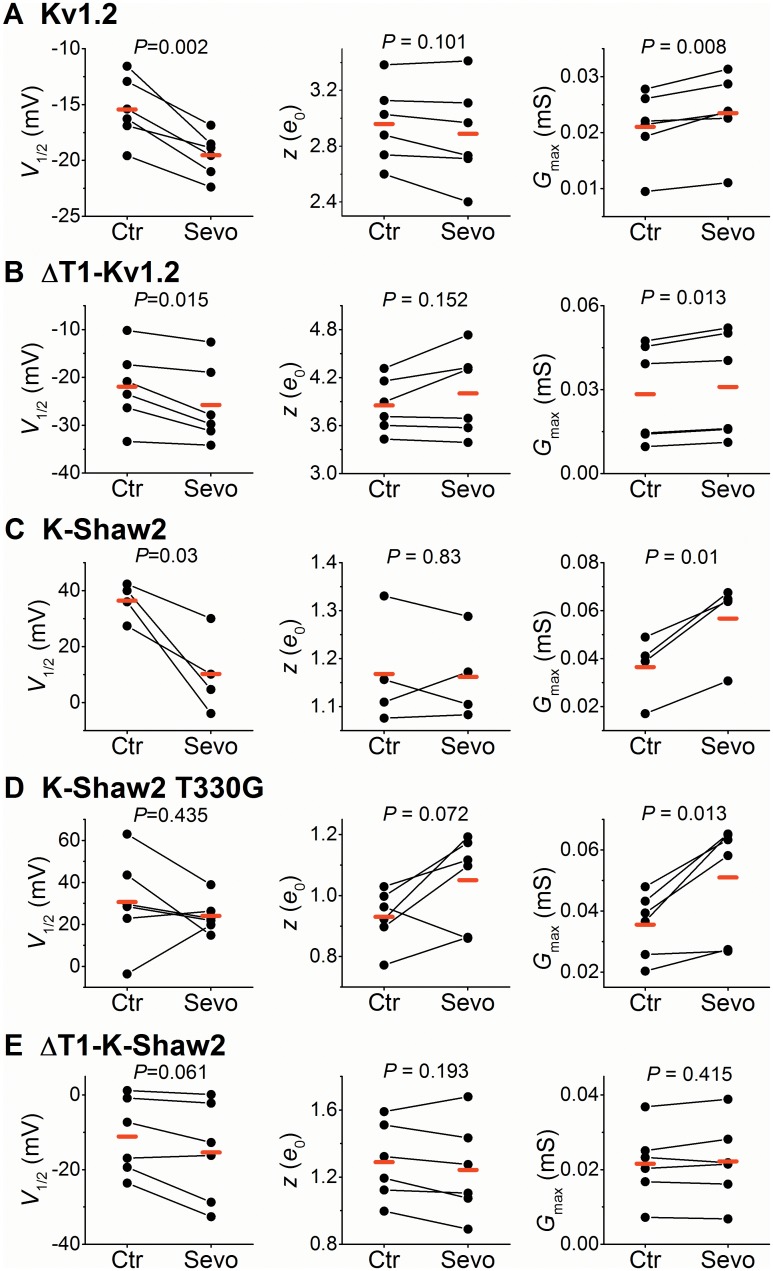
Analysis of *G*-*V* relations from Kv1.2, ΔT1-Kv1.2, K-Shaw2, K-Shaw2 T330G and ΔT1-K-Shaw2. (A) Best-fit Boltzmann parameters (*V*_1/2_, *z* and *G*_max_) from individual paired measurements before (Ctr) and after exposure to 1 mM sevoflurane (Sevo). Each pair of symbols connected by a *solid* line represents an individual paired experiment (Materials and Methods). The *G*_max_ graphs depict raw values before normalization (in mS). The *P* value resulting from a paired Student-*t* test is shown above each graph, and the *red* marks indicate the mean values of the sample. (B)–(E) are as described for panel A. The number oocytes examined for each Kv channel was 6, 6, 4, 6 and 6, respectively.

Previous studies showed that several heterologously expressed Kv1 channels are potentiated by anesthetic concentrations of sevoflurane [7, 9]. This modulation results primarily from shifting the *G*-*V* relation to the left and increasing the *G*_max_ (e.g., Kv1.2; Fig 1). Sevoflurane shifts the Kv1.2 *V*_1/2_ by -4 mV and increases the *G*_max_ by 13% (Figs 1A, 1B and 2A; Table A in S1 File). A close examination of the Kv1.2 *G*_Sevo_/*G*_0_ –voltage relation also shows a substantial sevoflurane-induced potentiation of the conductance at -30 mV (~180%) and a sustained potentiation at positive membrane potentials, which corresponds to the increase in *G*_max_ (Fig 1C). The potentiation at -30 mV is particularly significant because it is within the range where sevoflurane could affect action potential firing.

**Fig 3:**
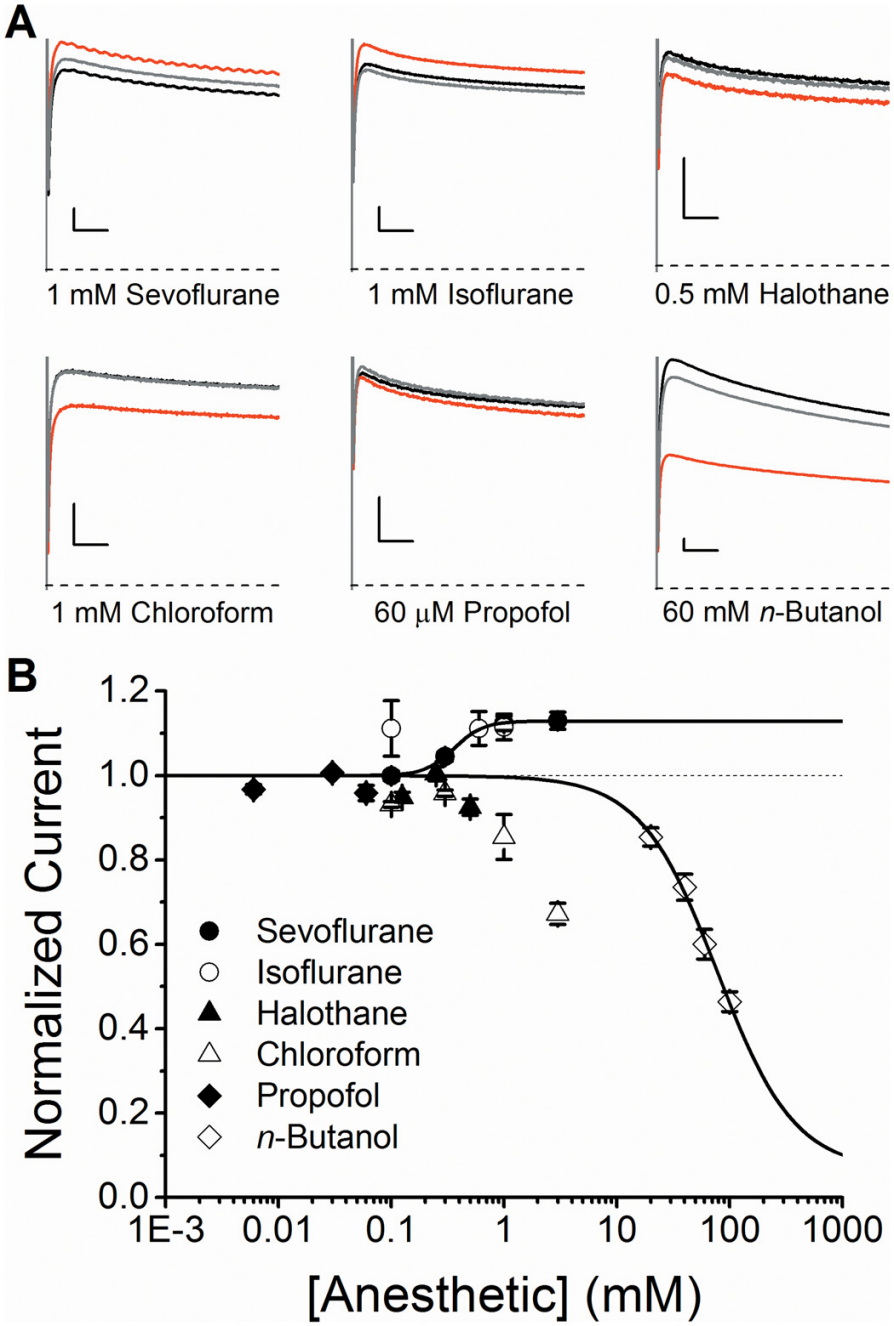
Modulation of the Kv1.2 channel by general anesthetics. (A) Effects of general anesthetics on the whole-oocyte Kv1.2 currents evoked by a voltage step to +60 mV from a holding voltage of -100 mV. *Black*, *red* and *grey* current traces correspond to control, anesthetic-exposed, and washout, respectively. The scale bars indicate 50 ms and 0.5 μA. (B) Concentration-response relations of various general anesthetics acting on the Kv1.2 channel. *Solid* lines are the best fits assuming the Hill equation for sevoflurane and *n*-butanol (Materials and Methods). Considering the magnitude of the change and the concentrations tested, only sevoflurane and *n*-butanol produced reliable Hill equation fits. N = 5–7 oocytes for each concentration. Best-fit parameters are summarized in Table 1.

To additionally screen isoflurane, halothane, chloroform, propofol and *n*-butanol, we tested their effects on the Kv1.2 current at +60 mV (Fig 3). Sevoflurane and isoflurane (1 mM) modestly potentiate the Kv1.2 conductance by 12.4 ± 1.7% and 11.5 ± 3.1%, respectively (Fig 3B). For sevoflurane, *K* and *n*_H_ are 360 μM and 3.3, respectively, which demonstrates relevant pharmacological potency and apparent cooperativity (Fig 3B and Table 1). Potentiation by isoflurane was already maximal at 100 μM and apparent binding parameters were not estimated. By contrast to sevoflurane and isoflurane, halothane, chloroform and propofol displayed only modest inhibitory effects, while high concentrations of *n*-butanol produced a more robust inhibition (Fig 3B). Focusing on sevoflurane, we noticed that the previously reported potentiation of K-Shaw2 at +60 mV is ~3-fold greater than that of Kv1.2 [7]. Based on earlier work [6], we hypothesized that differences in the linker connecting the voltage-sensing domain to the pore domain (S4-S5 linker) might explain the differential responses to sevoflurane.

### A single amino acid substitution in the Kv1.2 S4-S5 linker (G329T) confers dramatic potentiation by general anesthetics

**Fig 4:**
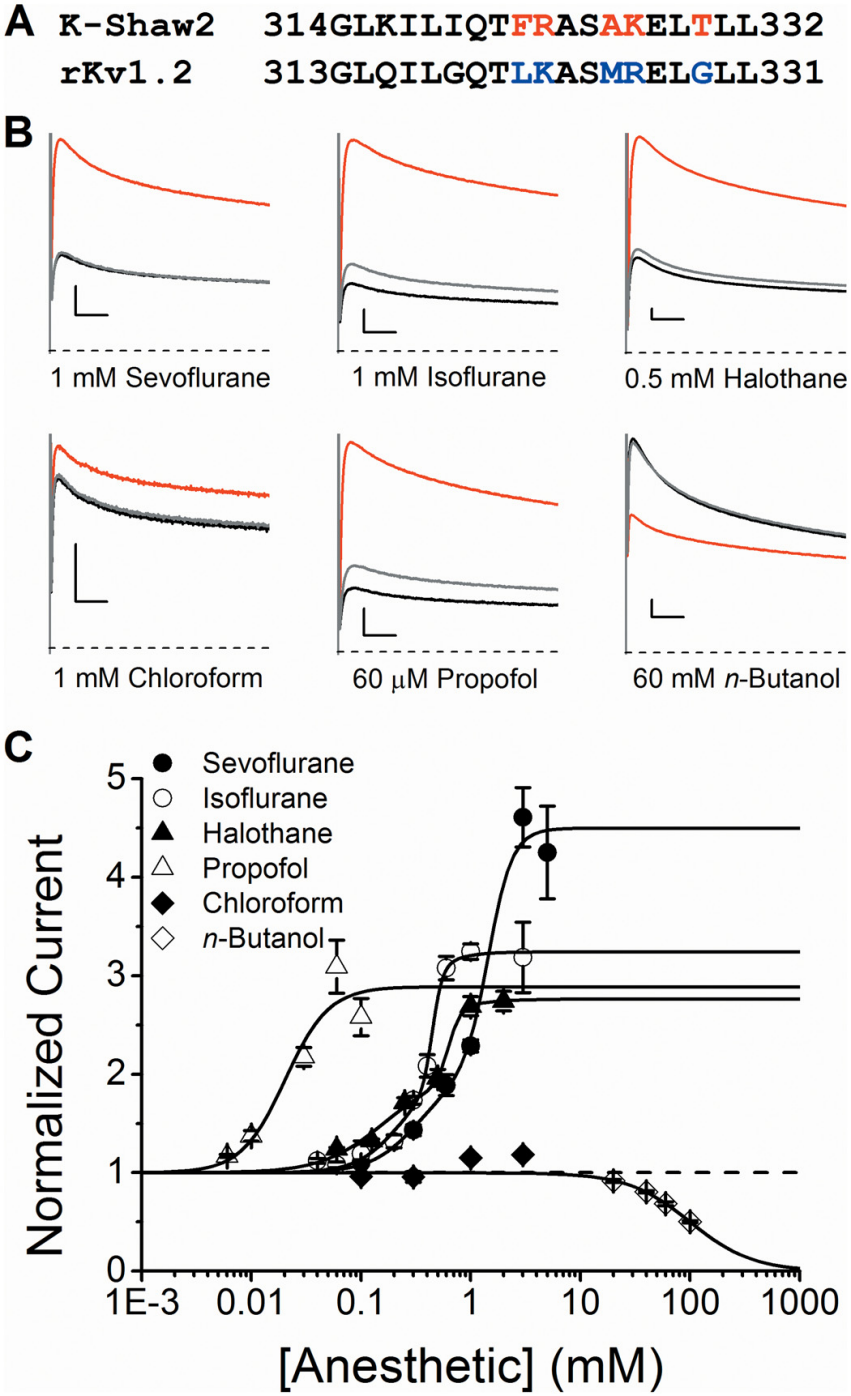
Modulation of Kv1.2 FRAKT by general anesthetics. (A) Sequence alignment of the S4-S5 linker from K-Shaw2 (314–332) and Kv1.2 (313–331) channels. Starting and ending residue numbers of the shown segments are indicated. In Kv1.2, the blue colored residues were swapped for the red colored residues in K-Shaw2 to create the Kv1.2 FRAKT mutant channel. (B) Effects of general anesthetics on whole-oocyte Kv1.2-FRAKT currents evoked by a voltage step to +60 mV from a holding voltage of -100 mV. *Black*, *red* and *grey* current traces correspond to control, anesthetic-exposed, and washout, respectively. The scale bars indicate 50 ms and 0.5 μA. (C) Concentration-response relations of various general anesthetics acting on the Kv1.2 FRAKT channel. *Solid* lines are the best fits assuming the Hill equation (propofol and *n*-butanol) or a double Hill equation (sevoflurane, isoflurane, and halothane) (Materials and Methods). Due to the small magnitude of the chloroform results, no reliable Hill equation fit could be obtained. N = 5–8 oocytes for each concentration. Best-fit parameters are summarized in Table 1.

To investigate the role of the S4-S5 linker in the modulation of Kv1.2 by general anesthetics, we focused on fourteen residues between positions 317 and 330 in K-Shaw2. Within the equivalent region in Kv1.2, there are six substitutions (Fig 4A). The Kv1.2-G319I mutation is, however, lethal (not shown). Therefore, to test the impact of the remaining differences, we converted the S4-S5 linker of Kv1.2 into that of K-Shaw2 between the corresponding Kv1.2 positions (residues 319–329) to create the Kv1.2-FRAKT chimera. Kv1.2-FRAKT currents evoked by a depolarizing step to +60 mV exhibit a dramatically different pattern of responses to various general anesthetics (Fig 4B and 4C). Sevoflurane, isoflurane, halothane, chloroform and propofol all potentiate the conductance to various degrees, while the inhibitory response to *n*-butanol remained unaffected. Also, the concentration-potentiation relations for sevoflurane, isoflurane and halothane exhibit complex profiles, which are reminiscent of the previously described potentiation of the K-Shaw2 channel by sevoflurane [7]. The concentration dependence of the potentiation is a biphasic phenomenon, occurring gradually at low concentrations and sharply at higher concentrations (Fig 4C). To empirically characterize this behavior, we assumed two classes of independent binding sites exhibiting (Materials and Methods; Fig 4C): 1) apparent high affinity and no cooperativity (*n*_H_ ≈ 1); and 2) apparent low affinity and highly cooperative interactions (*n*_H_ >>1). Although propofol is the most potent, sevoflurane exhibits the highest efficacy followed by isoflurane, halothane and chloroform (Fig 4C and Table 1). Maximum potentiation by propofol, isoflurane and halothane is, however, similar (2.7 to 3.3-fold above control; Table 1). Although the S4-S5 mutations conferred enhanced potentiation of Kv1.2 by sevoflurane that is similar to that previously observed with K-Shaw2, they also introduced novel potentiation by other general anesthetics. Thus, discrete structural changes in the S4-S5 linker can dramatically influence the ways in which general anesthetics interact with Kv channels. Since sevoflurane is clinically relevant and is the most efficacious positive modulator on Kv1.2-FRAKT and K-Shaw2, we focused on this anesthetic to dissect the structural basis of the potentiation.

**Fig 5:**
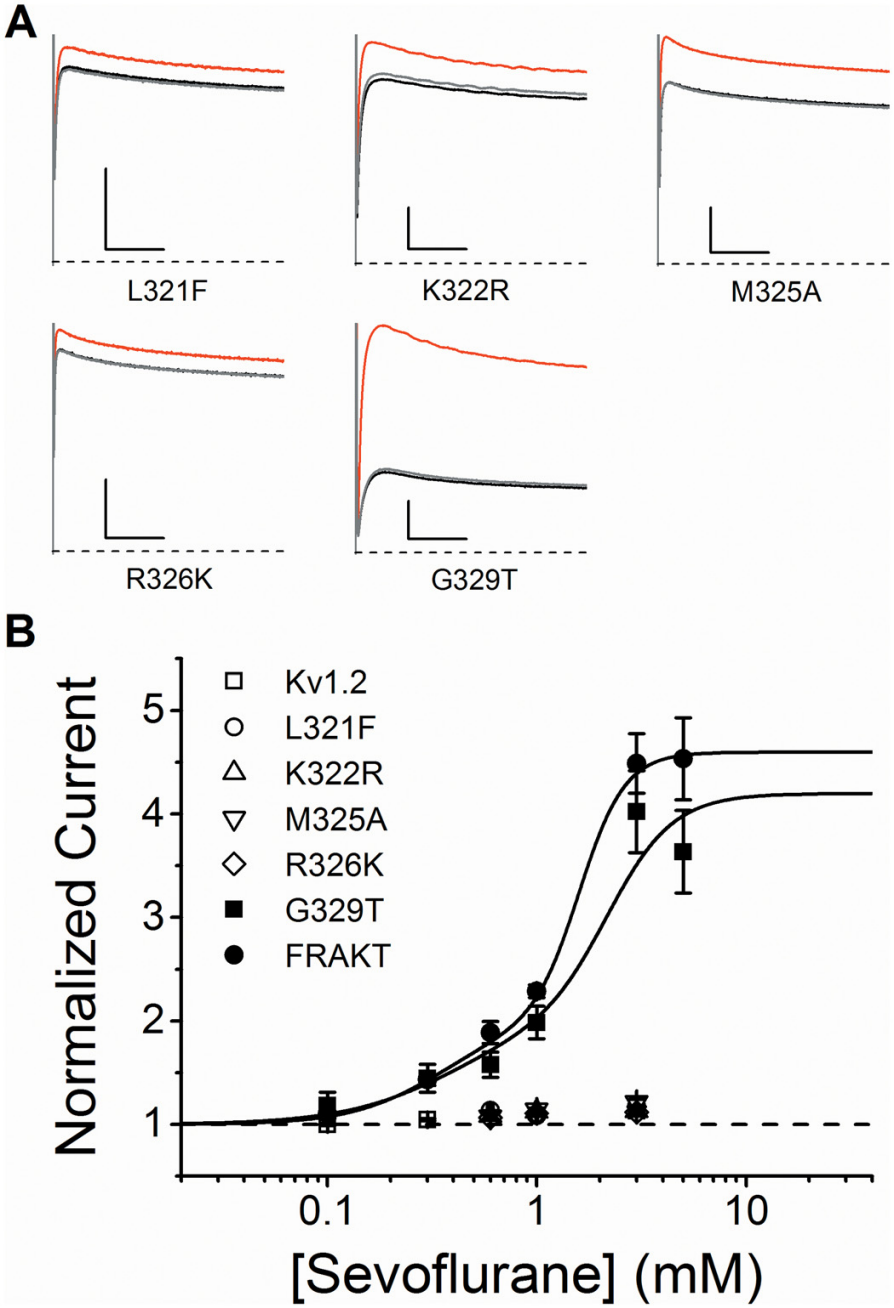
Kv1.2 G329T recapitulates the magnified positive modulation of Kv1.2 FRAKT by sevoflurane. (A) Effects of 1 mM sevoflurane on mutant whole-oocyte Kv1.2 currents evoked by a voltage step to +60 mV from a holding voltage of -100 mV. *Black*, *red* and *grey* current traces correspond to control, anesthetic-exposed, and washout, respectively. The scale bars indicate 50 ms and 1 μA. (B) Concentration-response relations of various general anesthetics acting on wild type and mutant Kv1.2 currents. *Solid* lines are the best fits assuming the double Hill equation (Materials and Methods). N = 4–8 oocytes for each dose. Best-fit parameters are summarized in Table 1.

To determine the specific amino acid(s) responsible for the dramatic sevoflurane potentiation of Kv1.2-FRAKT, we tested each substitution individually by creating the following Kv1.2 mutants: L321F, K322R, M325A, R326K and G329T. Whereas the potentiation of L321F, K322R, M325A, R326K is similar to that of wild type Kv1.2, G329T exhibited dramatic potentiation not significantly different from that of Kv1.2-FRAKT (two-way ANOVA, *P*>0.05; Fig 5 and Table 1). These results suggest that a single residue in the S4-S5 linker is a critical determinant of the potentiation of the Kv1.2 current by sevoflurane.

### Kv1.2-FRAKT and Kv1.2-G329T exhibit novel voltage dependent gating

**Fig 6:**
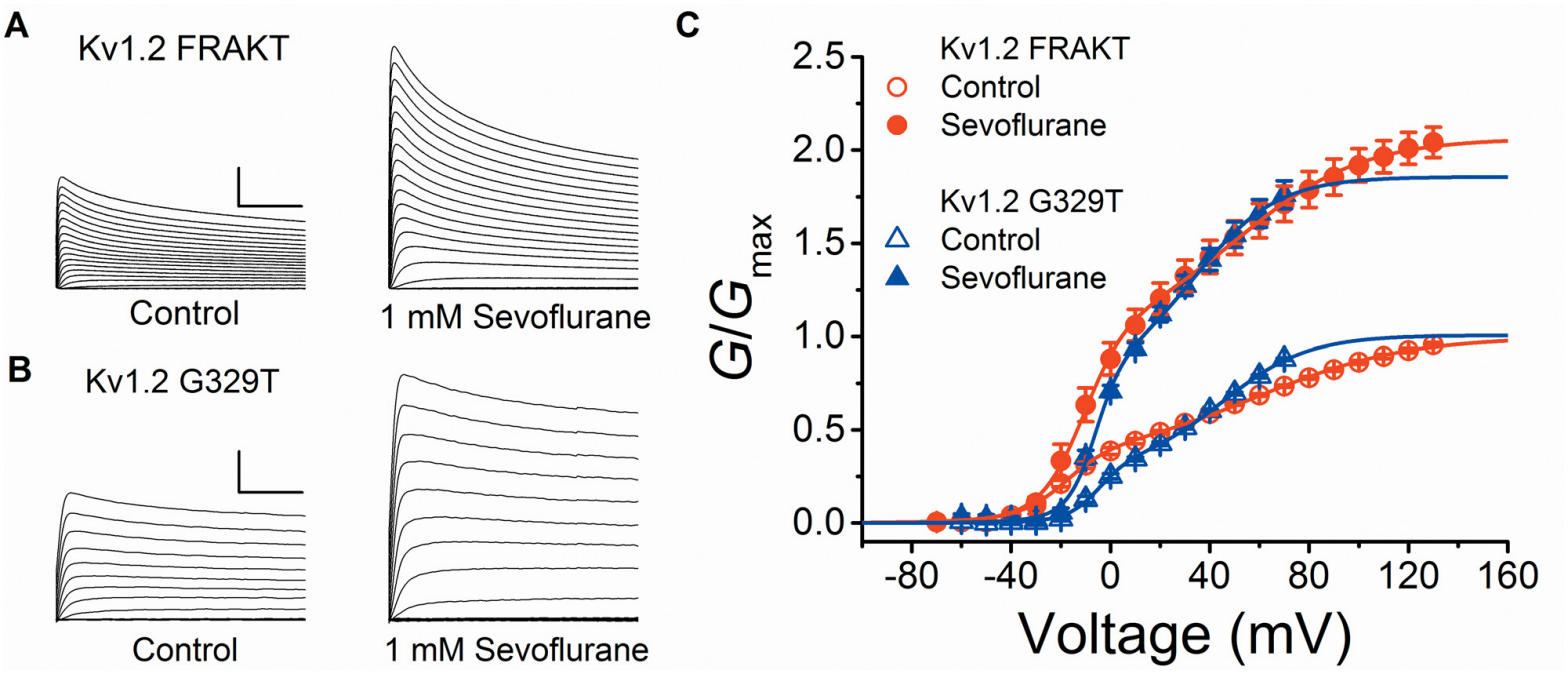
Novel *G*-*V* relations of Kv1.2 FRAKT and Kv1.2 G329T in the absence and presence of sevoflurane. (A) Families of whole-oocyte Kv1.2 FRAKT currents in the absence (*left*) and presence of 1 mM sevoflurane (*right*). Currents were evoked by step depolarizations from a holding voltage of -100 mV. The steps were delivered in increments of 10 mV from -90 to 130 mV. The scale bars indicate 100 ms and 2 μA. (B) Families of whole-oocyte Kv1.2 G329T currents in the absence (*left*) and presence of 1 mM sevoflurane (*right*). Currents were evoked by step depolarizations from a holding voltage of -100 mV. The steps were delivered in increments of 10 mV from -90 to 70 mV. The scale bars indicate 100 ms and 1 μA. (C) *G*-*V* relations of Kv1.2 FRAKT (*red*) and Kv1.2 G329T (*blue*) under control (*open*) or with 1 mM Sevoflurane (*filled*) (N = 6, 4, respectively). *Solid* lines are the best fits assuming a double Boltzmann equation (Materials and Methods). The best-fit parameters are summarized in Table A in S1 File.

The G329T mutation could have affected voltage-dependent gating and thereby allosterically (depending on conformation) influence the interaction of sevoflurane with the channel. Although the mutations do not appear to have gross effects on the overall current kinetics, Kv1.2–FRAKT and G329T dramatically remodel the *G*-*V* relation (Materials and Methods). Within a narrow range of membrane potentials (10–20 mV), the wild type Kv1.2 *G*-*V* relation sharply rises to the maximum conductance (*G*_max_) between +20 and +40 mV (Fig 1B). By contrast, the *G*-*V* relations of Kv1.2-FRAKT and Kv1.2-G329T rise to the *G*_max_ in a more gradual manner and exhibit two distinct components (Fig 6A). These *G*-*V* relations approach *G*_max_ at voltages > +100 mV and require the sum of at least two Boltzmann terms to adequately describe them (Fig 6B). Moreover, the *G*-*V* relations of Kv1.2-FRAKT and Kv1.2-G329T have similar profiles, albeit there are some quantitative differences (Fig 6; Table A in S1 File). For Kv1.2-FRAKT and Kv1.2-G329T: the median voltages (*V*_med_) are 22±2 and 28±2 mV, respectively (Fig 6). Relative to Kv1.2-FRAKT, Kv1.2-G329T modestly depolarizes *V*_1/2,1_ by ~13 mV and induces a 16 mV negative shift of *V*_1/2,2_ (Fig 6; Table A in S1 File). The corresponding equivalent gating charges (*z*_1_ and *z*_2_) increase from 2.7±0.1 to 4.8±0.1 e_0_, and from 0.9±0.1 to 1.5±0.1 e_0_ (Fig 6; Table A in S1 File). Nevertheless, among all individual FRAKT mutations, G329T is the only one that closely recapitulates the behavior of the Kv1.2-FRAKT indicating that it plays the most critical role in determining the novel gating phenotype of the chimera. Individual mutations of other residues (L321F, K322R, M325A, R326K) had little to no effect on Kv1.2 *G*-*V* relation (Fig A in S1 File).

**Fig 7:**
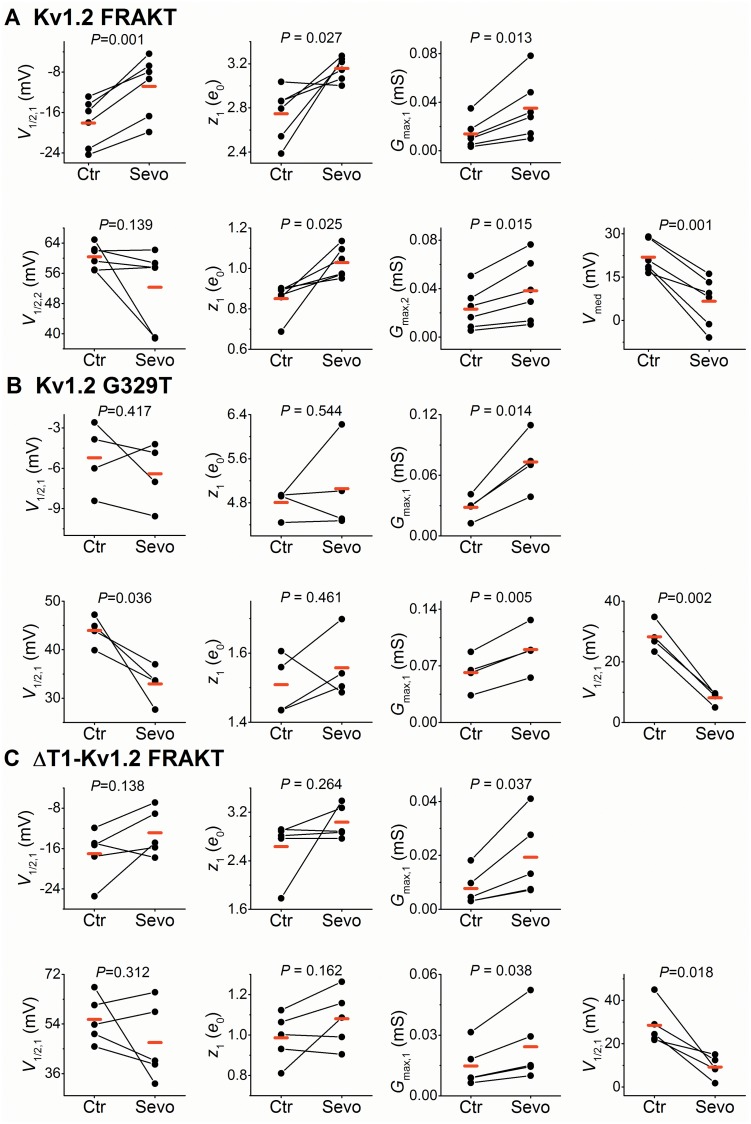
Analysis of bimodal *G*-*V* relations from Kv1.2 FRAKT, Kv1.2 G329T and ΔT1-Kv1.2 FRAKT. (A) Best-fit double Boltzmann parameters (*V*_1/2,1_, *z*_1_, *G*_max,1_, *V*_1/2,2_, *z*_2_, *G*_max,2_) and *V*_med_ from individual paired measurements before (Ctr) and after exposure to 1 mM sevoflurane (Sevo). Each pair of symbols connected by a *solid* line represents an individual paired experiment (Materials and Methods). The *G*_max_ graphs depict raw values before normalization (in mS). The *P* value resulting from a paired Student-t test is shown above each graph, and the *red* marks indicate the mean values of the sample. (B)–(C) are as described for panel A. The number oocytes examined for each Kv channel was 6, 4, 5, respectively.

Upon exposure to 1 mM sevoflurane, the *G*-*V* relations of Kv1.2-FRAKT and Kv1.2-G329T display changes that are qualitatively similar to those observed with Kv1.2 wild type; however, the net magnitudes are substantially larger (Fig 7A and 7B; Table A in S1 File). Δ*V*_med_ shifts to the left by ~15 and ~20 mV, respectively. On Kv1.2-FRAKT, sevoflurane induced small-modest changes in *V*_1/2,1_, *z*_1_ and *z*_2_; however, sevoflurane most noticeably doubles the total conductance (*G*_TOTAL_ = *G*_max1_+*G*_max2_ = 2.07) as a result of increasing *G*_max1_ (0.37 to 0.99) and *G*_max2_ (0.63 to 1.07) (Fig 7A; Table A in S1 File). Kv1.2-G329T, in contrast, displayed more selective effects on the bimodal *G-V* relation. The *V*_1/2,1_ and *z*_1_ are not affected, whereas the sevoflurane-induced negative Δ*V*_1/2,2_ is substantial (-11 mV, with no change in *z*_2_). Additionally, as found with Kv1.2-FRAKT, sevoflurane nearly doubles the total Kv1.2-G329T conductance (*G*_TOTAL_ = 1.89) (Fig 7B; Table A in S1 File). These results demonstrate that G329T alone markedly alters gating and concomitantly enhances the potentiation by sevoflurane, suggesting a strong link between voltage-dependent gating and anesthetic action.

### K-Shaw2-T330G eliminates the sevoflurane-induced voltage-dependent shift without affecting the sevoflurane-induced increase in *G*_max_

**Fig 8:**
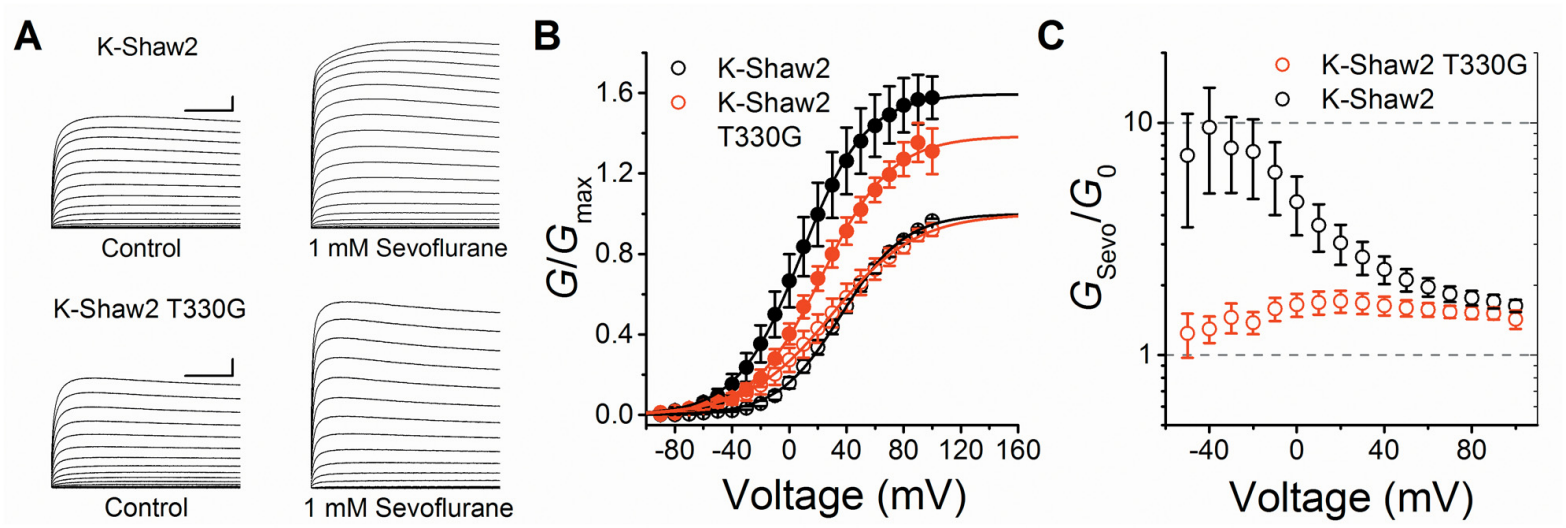
The T330G mutation eliminates the voltage-dependent potentiation of the K-Shaw2 conductance by sevoflurane. (A) Families of whole-oocyte K-Shaw2 (N = 4) and K-Shaw2 T330G (N = 6) currents in the absence (*left*) and presence of 1 mM sevoflurane (*right*). Currents were evoked by step depolarizations from a holding voltage of -100 mV. The steps were delivered in increments of 10 mV from -90 to +100 mV. The scale bars indicate 100 ms and 1 μA. (B) *G*-*V* relations of K-Shaw2 (*black*) and K-Shaw2 T330G (*red*) in the absence (*open*) and presence of 1 mM sevoflurane (*filled*). *Solid* lines are the best-fits to the Boltzmann equation. Best-fit parameters are summarized in Fig 2 and Table A in S1 File. (C) The voltage dependence of the conductance ratio (*G*_Sevo_/*G*_0_) K-Shaw2 (*black*) and K-Shaw2 T330G (*red*).

Since G329 in Kv1.2 critically controls voltage-dependent activation and keeps positive modulation by sevoflurane in check, we asked whether the reverse mutation in K-Shaw2 (T330G) would inhibit the naturally robust potentiation of this Kv channel by sevoflurane. In contrast to Kv1.2-G329T, K-Shaw2-T330G does not significantly affect the *G*-*V* relation (Fig 2; Table A in S1 File); however, it abolishes the sevoflurane-induced leftward shift of the *G*-*V* relation (Figs 2, 8A and 8B; Table 1; Table A in S1 File). Accordingly, T330G dramatically inhibits the potentiated conductance ratio (*G*_Sevo_/*G*_0_) from ~10 to ~1.3 at -40 mV (Fig 8C). However, the sevoflurane-induced increase in the *G*_max_ of K-Shaw2-T330G and wild type K-Shaw2 are very similar (1.4- and 1.6-fold, respectively; Table A in S1 File). Accordingly, the *G*_Sevo_/*G*_0_ of wild type and mutant channels converge toward a similar value at +100 mV (~1.5; Fig 8C). This result suggests that the positive modulation induced by sevoflurane has two separable components. One depends on the role of a specific residue in the S4-S5 linker that controls voltage-dependent gating, whereas the other is independent of that residue and namely affects the *G*_max_.

### The role of the T1 domain on the modulations of Kv1.2, Kv1.2-FRAKT and K-Shaw2 by sevoflurane

**Fig 9:**
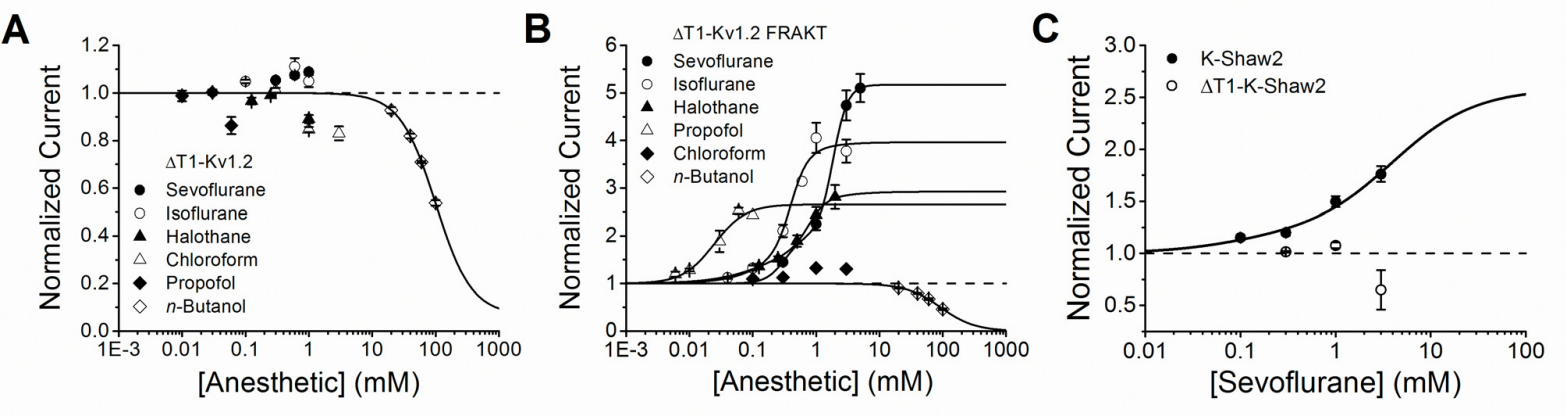
Positive modulation by sevoflurane is T1 domain-independent in Kv1.2 and Kv1.2-FRAKT, and T1 domain-dependent in K-Shaw2. (A) Concentration-response relations of various general anesthetics acting on ΔT1-Kv1.2. *Solid* line is the best fit to the Hill equation for *n*-butanol. (B) Concentration-response relations of various general anesthetics acting on ΔT1-Kv1.2 FRAKT. *Solid* lines are the best fits to the Hill equation (propofol and *n*-butanol) or double Hill equation (sevoflurane, isoflurane, and halothane). Best-fit parameters for results in panels A and B are summarized in Table 1. (C) Concentration-response relations of sevoflurane acting on K-Shaw2 and ΔT1-K-Shaw2. *Solid* line is the best-fit double Hill equation to the K-Shaw2 data with the following parameters: *K*_1_ = 0.08 mM, *A*_1_ = 0.18, *n*_H1_ = 1, *K*_2_ = 4 mM, *A*_2_ = 1.4, *n*_H2_ = 1. These parameters are similar to those previously published for wild type K-Shaw2 (Table 1) [7]. K-Shaw2 and ΔT1-K-Shaw2 were tested at +60 mV. N = 2–8 oocytes for each dose.

Previous studies strongly suggest that the intracellular regulatory T1 domain of Kv channels plays a significant role in gating [24–28]. Thus, we asked whether the T1 domain might also contribute to the positive modulation of Kv1.2, Kv1.2-FRAKT and K-Shaw2, by sevoflurane. In ΔT1-Kv1.2 and ΔT1-Kv1.2-FRAKT, residues 2–122 are missing; and ΔT1-K-Shaw2 lacks residues 2–155. ΔT1-Kv1.2 and ΔT1-Kv1.2-FRAKT exhibited *G*-*V* relations and responses to general anesthetics that are similar to those from their full-length counterparts (Figs 2B, 7C, 9A and 9B; Fig B and Table A in S1 File). Sevoflurane shifted the ΔT1-Kv1.2 *V*_1/2_ by -4 mV, and increased the *G*_max_ by 10%. It also shifted the ΔT1-Kv1.2-FRAKT *V*_med_ by -19 mV and nearly doubled the *G*_TOTAL_ (1.9-fold). By contrast, ΔT1-K-Shaw2 exhibited a substantially leftward shifted *G-V* relation (-48 mV; data not shown) and no positive effect of sevoflurane on *V*_1/2_ and *G*_max_ (Fig 2; Table A in S1 File). These results suggest that voltage-dependent gating of Kv1.2 and Kv1.2-FRAKT and their modulation by general anesthetics are independent of the T1 domain. K-Shaw2 gating and its positive modulation by sevoflurane are, however, completely dependent on the presence of the T1 domain, suggesting specialized interactions between the T1 domain and the gating apparatus of the K-Shaw2 channel. Thus, we can infer that the dual positive modulation of the Kv1.2 channel by sevoflurane (leftward shifted *G*-*V* relation and increased *G*_max_) only implicates regions directly involved in gating (the voltage-sensing and pore domains). Based on this outcome, we created minimal atomic models of ΔT1-Kv1.2 and ΔT1-Kv1.2 G329T to explore putative *in-silico* interaction sites in the transmembrane regions of the channel.

### Putative sevoflurane binding sites in ΔT1-Kv1.2 and ΔT1-Kv1.2-G329T

The results so far demonstrate that 1) sevoflurane potentiates the Kv1.2 current with relevant apparent affinity and cooperativity and is independent of the T1 domain; 2) sevoflurane interacts with two independent classes of sites in K-Shaw2, Kv1.2-FRAKT and Kv1.2-G329T (with apparent high and low affinities); 2) G329T is sufficient to confer dramatic positive modulation by sevoflurane and other general anesthetics; 3) the potentiation of Kv1.2-G329T by sevoflurane resembles the action of this anesthetic on K-Shaw2; 4) G329T fully recapitulates the electrophysiological and pharmacological phenotype of the Kv1.2-FRAKT chimera, which is also independent of the T1 domain; 5) the reverse K-Shaw2-T330G mutation at a position equivalent to that of G329 in Kv1.2 nearly eliminates the sevoflurane-induced leftward shift of the K-Shaw2 *G*-*V* relation. These results suggest that G329 in the S4-S5 linker plays a key role controlling the potentiation of the Kv1.2 current by sevoflurane. In the light of these observations, we explored putative sites of sevoflurane action in the Kv1.2 channel to help elucidate a working mechanism of anesthetic action. To identify putative sevoflurane binding sites with atomic resolution, we applied docking and MD-based free-energy calculations. Specifically, we docked sevoflurane against an ensemble of 120 membrane-equilibrated structures of ΔT1-Kv1.2 and ΔT1-Kv1.2-G329T in the activated-open and resting-closed conformations (Materials and Methods and S1 File). The sampling of an ensemble accounts for the molecular flexibility of the protein. Then, starting from sevoflurane-bound channel structures, we carried out free-energy calculations using the LIE method to resolve site-specific affinities of sevoflurane (S1 File). This calculation assumes that individual sites are independent. Also, because electrophysiological experiments do not measure actual binding of the anesthetics to the Kv channels, we made no attempt to quantitatively compare an “apparent” affinity (electrophysiological estimate) against the calculated affinities of multiple putative sites (LIE estimates). To learn about relative properties of putative sites, we only compared calculated affinities between different classes of sites in putative resting-closed and activated-open conformations.

**Fig 10:**
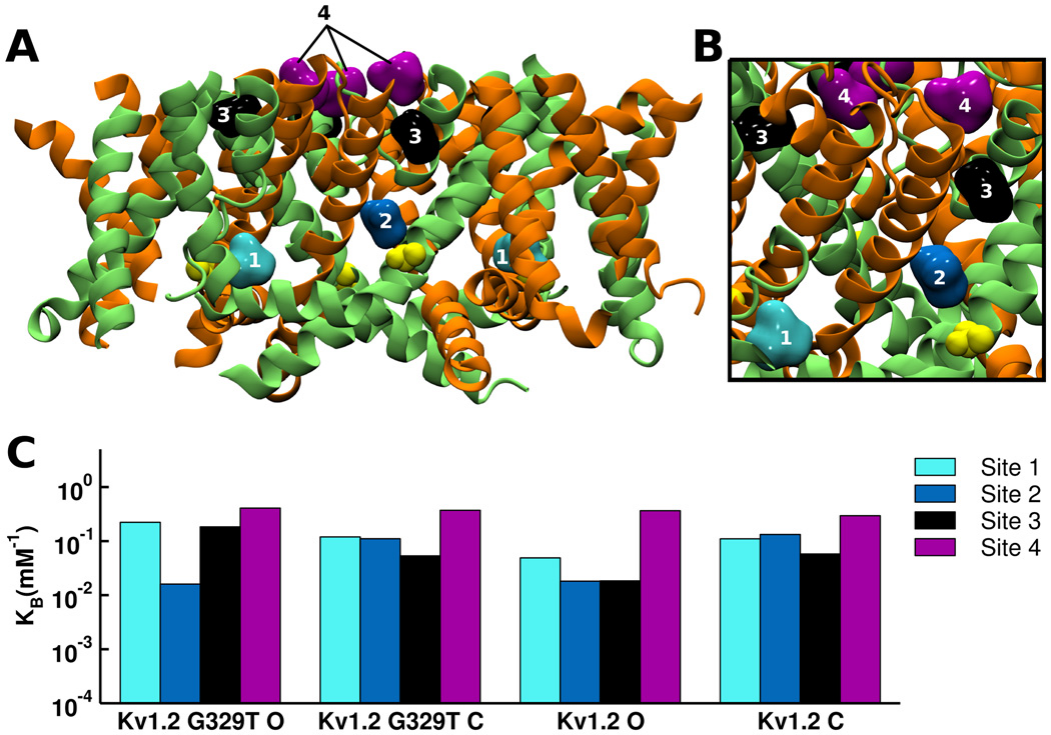
Putative sevoflurane binding sites in the Kv1.2 channel. (A) Representation of four distinct sevoflurane binding locations on Kv1.2: site 1 (*light blue*), 2 (*blue*), 3 (*black*) and 4 (*purple*). Each pair of subunits is represented in *green* and *orange*. Mutation G329T is highlighted yellow. (B) Close-up view of ligand binding sites and mutation. Note that site 2 is in close proximity to the mutated residue G329T. (C) Binding constants of individual sevoflurane sites. These estimates were obtained using the LIE method as described under S1 File. O and C stand for activation-open and resting-closed conformations of the channel.

Sevoflurane is found at four distinct locations on the Kv channel structures, hereafter called sites 1–4 (Fig 10A). Sites 1 and 2 are located near the S4-S5 linker at the internal face of the channel; site 3 is formed in the S5-S6 interface of adjacent subunits; and site 4 involves discrete pockets around the selectivity filter at the extracellular face of the channel. Sites 1 and 2 are near G329T in the mutant channel (Fig 10B). Despite a similar pattern of binding sites in Kv1.2 and Kv1.2-G329T, the LIE analysis of interaction energies revealed subsets of non-overlapping sites with relatively low and high affinities (Fig 10C; Table B in S1 File). Sites 1–3 involving the S4-S5 linker and pore domain exhibit relatively low-affinities with binding constants ranging between 0.01–0.2 mM^-1^; and site 4 around the selectivity filter exhibits relatively high-affinity ranging between 0.3–0.4 mM^-1.^ In addition, the binding constants of sites 1 and 3 display modest conformation dependence, with a slight preference for the open state in Kv1.2-G329T. In contrast, the site 2 binding constants in the resting-closed conformation are ~7-fold greater than in the fully open conformation, for both wild type Kv1.2 and Kv1.2-G329T. Site 4 exhibits the largest binding constants, but no conformation dependence (Fig 10C; Table B in S1 File). These results are consistent with a scenario in which the modulation of Kv1.2 channels by sevoflurane results from multiple interactions with distinct classes of sites. A subset of these sites is possibly responsible for leftward shifting the *G*-*V* relation and another potentially contributing to the net increase in *G*_max_. However, since the calculated binding constants of wild type Kv1.2 and Kv1.2-G329T are generally similar (ranging between 0.02–0.4 mM^-1^), additional factors probably determine the larger efficacy of sevoflurane acting on the mutant channel.

## Discussion

### Distinct gating processes in Kv channels

Shaker-related Kv1 channels undergo complex and strongly voltage-dependent activation gating involving sequential transitions between multiple closed states and a final cooperative opening step [29–31]. This mechanism has two major steps. The first corresponds to a major conformational change of the voltage sensors, which unlocks the gating machinery; and the second corresponds to a final cooperative rearrangement of the voltage sensors, which ultimately opens the intracellular activation gate [32, 33]. Whereas the first step is associated with movement of a large gating charge (14–16 e_0_), the second only involves movement of a small gating charge (~1 e_0_). Therefore, tightly coupled voltage-dependent gating is typically dominated by the first component. The previously investigated Shaker-ILT voltage sensor mutant clearly unveiled the second step by greatly shifting its voltage dependence toward depolarized voltages [32, 33]. The K-Shaw2 channel is a special case where the weakly voltage-dependent component is sufficient to control activation gating and can be described as a simple first-order equilibrium [34, 35]. Here, we showed that mutations that converted the S4-S5 linker of Kv1.2 into that of K-Shaw2 exhibit a Shaker-ILT-like gating phenotype, albeit the separation of the two activation components is further exaggerated. These mutations do not have much effect on the first sharp component of the *G*-*V* relation (Table A in S1 File). In contrast, the second gating component of the mutant *G*-*V* relations becomes clearly evident as a result of a robust depolarizing shift of its voltage dependence. Remarkably, the profile and voltage dependence of this depolarized component resembles the K-Shaw2 *G*-*V* relation. We propose that the G329T mutation at the C-terminal end of the S4-S5 linker induces these changes by greatly reducing the weakly voltage-dependent equilibrium constant of the cooperative opening transition. Such a reduction could directly result from hampering protein backbone flexibility caused by the glycine to threonine substitution at a critical position. The flexibility conferred by glycine at this position might create a critical pivot point generally conserved in a majority of Kv channels (Fig C in S1 File). The rare natural substitution of glycine for threonine at the equivalent position in wild type K-Shaw2 might help determine the unusual gating properties of K-Shaw2 and its distinctive strong positive modulation by sevoflurane [7].

### Putative mechanism of sevoflurane action on Kv channels

The positive modulation of Kv channels by sevoflurane is associated with two separable effects. One shifts the *G*-*V* relation leftward and the other increases the *G*_max_. Sevoflurane might thus simultaneously influence two separable processes by acting at two distinct sites. We propose that sevoflurane shifts the *G*-*V* relation leftward by having a positive influence on the final weakly voltage-dependent component of gating that ultimately controls the opening of the Kv channel. The anesthetic might achieve this by interacting with the S4-S5 linker and the pore domain. The Kv channel’s S4 voltage sensor transmits its final concerted conformational change to the S4-S5 linker, which in turn acts on the intracellular activation gate to open it [14, 15, 18]. This idea helps explain quantitatively distinct sevoflurane modulations of Kv1.2 and K-Shaw2 channels and how S4-S5 linker mutations have profound effects on the final gating component and the positive modulation by sevoflurane and other general anesthetics. K-Shaw2 gating is dominated by the concerted conformational change transmitted by the S4-S5 linker, (in contrast to more conventional gating in Kv1 channels; see section above), and sevoflurane might selectively influence this process. Therefore, sevoflurane appears to have a greater effect on the voltage dependence of K-Shaw2 than on that of Kv1.2. However, upon introducing the G329T mutation at the C-terminal end of the Kv1.2 S4-S5 linker, the final gating component appears rightward shifted and magnified. Consequently, the potentiation of the Kv1.2-G329T by sevoflurane is also magnified, which seems to namely result from selectively leftward shifting the final gating component. To cause this shift, sevoflurane might increase the equilibrium constant of the opening step by destabilizing the pre-open closed state, as previously proposed for the wild-type K-Shaw2 [6] (see below). Supporting these ideas further, the reverse K-Shaw2 T330G mutation conversely eliminates the sevoflurane-induced leftward shift of the *G*-*V* relation, albeit has no significant effect on voltage-dependent gating (Fig 8). Additionally, this experiment surprisingly revealed that T330 is only a critical determinant of the sevoflurane effect on voltage dependence because the T330G mutation did not significantly affect the sevoflurane-induced increase in *G*_max_. This selectivity strongly suggests that another site is independently responsible for the sevoflurane-induced increase in the *G*_max_. Given the quick onset and reversibility of the positive modulation [7], we propose that, rather that increasing the number of functional channels, sevoflurane probably increases the maximum open probability and/or the unitary conductance. The potentiation of the ShakerB Kv channel by isoflurane results from the combination of both effects [36]. Further work is needed to investigate the biophysical and molecular underpinnings of the sevoflurane-induced increase in *G*_max_.

### Exploring putative sevoflurane binding sites and mechanisms of action in the Kv1.2 channel

We gained more specific insights into sites and mechanisms of sevoflurane action from atomic level simulations of a membrane-equilibrated Kv1.2 model in the resting closed and open states. In silico docking of sevoflurane to these conformations, along with free-energy calculations, revealed putative sites with distinct binding affinities in regions experimentally implicated in anesthetic action by the comparative study of the Kv1.2 and K-Shaw2 channels. Sites 1 and 2 are in close proximity to the S4-S5 linker. Sevoflurane binding to site 2 in Kv1.2 and Kv1.2-G329T is, however, significantly weaker in the open state than in the resting closed state (~7-fold). Thus, a simple stabilization of the open state alone does not explain the sevoflurane-induced leftward shift in the *G*-*V* relation. Rather, this shift might result from the crosstalk among various interacting sites, which globally destabilizes the pre-open closed state. Further modeling of sevoflurane binding sites in the pre-open closed state will be necessary to examine this possibility.

Why does Kv1.2-G329T exhibit greater sevoflurane-induced potentiation than the wild type counterpart? The G329T mutation alone might stabilize the pre-open closed state and consequently the voltage dependence of the opening step is better resolved as a separate depolarized gating component of the *G*-*V* relation (see above). That is, actual opening of the channel becomes harder. If sevoflurane binds to a pre-open closed state displaying an increased presence in the G329T mutant, it might have greater influence on its stability. Presumably, binding of sevoflurane to interacting sites in the tetrameric Kv1.2-G329T opposes the negative effect of the mutation by destabilizing the pre-open closed state, which results in the observed sevoflurane-induced leftward shift of the *G*-*V* relation’s second gating component. Essentially, sevoflurane might act similarly on wild type Kv1.2. In this case, however, the tight coupling between voltage-dependent activation and channel opening blurs the separation of the gating components in the *G*-*V* relation. This implies that, relative to the G329T, the relative availability of binding sites in the pre-open closed state of wild type Kv1.2 is more limited. Therefore, the dynamic range for positive modulation of wild type Kv1.2 by sevoflurane is more restricted and the potentiation less efficacious.

Sharply in contrast to sites 1–3, site 4 located externally around the selectivity filter displays the strongest binding constants (on average, ~5-fold stronger than the highest affinity of site 2), but little state dependence. Binding of sevoflurane to this site is particularly interesting because it might affect the stability of the selectivity filter, which plays a role in a mechanism of Kv channel inactivation [37]. Stabilization of the open conducting conformation of the selectivity filter following sevoflurane binding to site 4 could help explain the sevoflurane-induced increase in the *G*_max_. Further experimental validation would, however, be necessary to test this hypothesis. Multiple binding sites with distinct binding constants is consistent with the presence of low- and high-affinity components of the Kv1.2-FRAKT and Kv1.2-G329T concentration-potentiation relations for various inhaled anesthetics (Figs 4 and 5). However, no quantitative relationships could be made at this time.

Surprisingly, the positive modulation of K-Shaw2 by sevoflurane is T1 domain-dependent, whereas that of Kv1.2-FRAKT is not. This observation suggests that other interactions contribute to the ability of sevoflurane to influence the final opening step. Supporting this idea, the T1 domain scaffolding of Kv1.2 and K-Shaw2 channels exhibit significant differences in regions that have been implicated in gating [28, 38].

## Concluding remarks

Three major mechanistic conclusions emerge from this study. First, the positive modulation of Kv1.2 channels by general anesthetics depends on a single S4-S5 linker residue (G329). G329T confers dramatic positive modulation by sevoflurane Threonine is the residue naturally found at the equivalent position in the K-Shaw2 (T330), a Kv channel that normally exhibits strong positive modulation by sevoflurane. Underscoring the critical role of T330, the converse mutation T330G abolished voltage-dependent potentiation of K-Shaw2 by sevoflurane without affecting the sevoflurane-induced increase in *G*_max_. Such a differential effect suggests separable sites of sevoflurane action, which is consistent with modeling results. The location of G329 and T330 in the S4-S5 linker of these Kv channels suggests that this position is a critical pivot point of voltage-dependent gating. Second, depending on Kv channel specific interactions, the cytoplasmic T1 domain may additionally contribute to the positive modulation of K-Shaw2 by sevoflurane. Third, molecular modeling suggests that positive modulation results from allosteric interactions between sites 1–3, the pore domain and the S4-S5 linker -a critical moving part involved in activation gating. These interactions might favorably influence pore opening. Additionally, binding to site 4 might stabilize the open state. These mechanisms serve as framework to explain how Kv1 channels might play a role in general anesthetic action.

## Supporting Information

S1 File Liang et al Supporting Information. (DOCX)
