# Supplementary material for: Positive Allosteric Modulation of Kv Channels by Sevoflurane: Insights into the Structural Basis of Inhaled Anesthetic Action
Source: PLoS One. 2015 Nov 24;10(11):e0143363. doi: 10.1371/journal.pone.0143363 (PMC4657974; doi:10.1371/journal.pone.0143363)
Supplement: S1 File — (DOCX) [file pone.0143363.s001.docx]

**SUPPORTING INFORMATION**

**Positive allosteric modulation of Kv channels by sevoflurane: *insights into the structural basis of inhaled anesthetic action***

Qiansheng Liang, Warren D. Anderson, Shelly T. Jones, Caio S. Souza, Juliana M. Hosoume, Werner Treptow, and Manuel Covarrubias

**Supplemental Materials and Methods**

**Membrane Equilibrated Channel Structures**. Channel structures were embedded in the lipid bilayer for MD relaxation and subsequent molecular docking of sevoflurane. Specifically, the structures were inserted in a fully hydrated and neutral (zwitterionic) all atom palmitoyloleylphosphatidylcholine (POPC) phospholipid bilayer. Then, all systems were simulated over an MD simulation spanning ~ 20 ns, at constant temperature (300 K) and pressure (1 atm), neutral pH, and with no applied transmembrane (TM) electrostatic potential. As presented in Figure D, the channel structures remained stable in their starting resting-closed or activated-open conformations throughout the simulations. In the Kv1.2-G329T simulations, the root mean-square deviation (RMSD) values for the whole TM domain, as well as for segments S1–S6 (Pore) and the S4-S5 linker, range from 1.0 to 2.5 Å, which agrees with the structural drift quantified in control simulations of the wild-type channel.

**Docking Calculations.** Sevoflurane molecules were docked on each channel structure. Given that receptor flexibility influences ligand binding, anesthetics were docked against an ensemble of equilibrium channel structures generated via MD runs. Each ensemble consisted of 120 structures sampled over the final 6 ns of simulation. Docking solutions were clustered into sites according to their specific location on the channel structure. Using *AutoDock Vina* (Trott and Olson, 2010), a total of 1200 independent docking calculations were performed. Each docking calculation grid was adjusted to comprise the target protein pore, since it is directly associated with the gating process. Solutions with RMSD < 0.5 Å were considered to be the same. In all docking calculations the exhaustiveness parameter was set to 200 and ligands were allowed to have flexible bonds. A total of 240,000 solutions were obtained (6000 solutions per protein).

**Linear Interaction Energy Calculations.** In the Linear Interaction Energy (LIE) method (Aqvist et al., 1994), the binding free energy of ligand L to receptor R is given by

$\Delta G_{bind}= \alpha\left( \left\langle V^{vdW} \right\rangle_{bound}- \left\langle V^{vdW} \right\rangle_{free} \right)+ \beta(\left\langle V^{elect} \right\rangle_{bound}-\left\langle V^{elect} \right\rangle_{free}+ \gamma$ [1]

where, $\left\langle V^{vdW} \right\rangle$ and $\left\langle V^{elect} \right\rangle$ are ensemble averages of the van der Waals (vdW) and electrostatic (elect.) interaction potentials of the ligand, in its receptor-bound and solution-free states. The empirical parameters α and β are respectively scaling factors for the van der Waals and electrostatic interaction energies of the ligand, whereas γ is a constant free-energy term related to its solvation energy. In eq. [1], the non-bonded interaction potentials $V(\boldsymbol{X})$ are explicit functions of the microscopic configuration of the system, which allows for direct estimation of $\left\langle V^{vdW} \right\rangle$ and $\left\langle V^{elect} \right\rangle$ from MD-generated ensembles of each of the ligand reference states. In detail,

$V\left( \boldsymbol{X} \right)=V_{AB}\left( \boldsymbol{X} \right)\boldsymbol{-}V_{A}\left( \boldsymbol{X} \right)- V_{B}(\boldsymbol{X})$ [2]

with $V_{A}\left( \boldsymbol{X} \right)$ and $V_{B}(\boldsymbol{X})$ describing the contributions of the ligand and its environment to the total energy of the system $V_{AB}\left( \boldsymbol{X} \right)$.

Accordingly, for every Kv1.2 construct, the ensemble averages of sevoflurane, $\left\langle V^{vdW} \right\rangle_{free}$ and $\left\langle V^{elect} \right\rangle_{free}$ were estimated from an equilibrium MD trajectory of the ligand in its reference aqueous environment. Ensemble averages were directly evaluated by time averaging eq. [2] throughout the simulation. Still, the energy averages $\left\langle V^{vdW} \right\rangle_{bound}$ and $\left\langle V^{elect} \right\rangle_{bound}$ were computed from MD simulations of the ligand-bound channel as resolved from docking (Figure E). To ensure proper sampling of the bound ensemble, the molecular system was simulated following a perturbed Hamiltonian $H^{*}\left( X, r \right)= H\left( X \right)+h(r)$ depending further on the separation distance of the ligand from the binding site. Here, *H*(*X*) is the original Hamiltonian of the system and $h\left( r \right)= k/2\left( r-r^{*} \right)^{2}$, an external potential that biases the ligand towards the bound state ($r=r^{*}$) with a force constant $k$. Under this scheme, unbiased averages $\left\langle V \right\rangle$were computed from eq. [2] according to

$\left\langle V \right\rangle= \frac{\left\langle\frac{V}{e^{-\beta h\left( r \right)}} \right\rangle_{*}}{\left\langle\frac{1}{e^{-\beta h\left( r \right)}} \right\rangle_{*}}$ [3]

with $\left\langle\right\rangle*$ denoting ensemble averages over the biased probability distribution as generated from the perturbed MD simulation.

The site-specific binding free energies of sevoflurane as presented in Table B were then obtained by plugging the ensemble energy averages into eq. [1]. Here, the empirical parameters $\alpha$ and $\beta$ were respectively set as 0.18 and 0.34 since they were proved to recreate experimental binding free energies successfully for a variety of ligand-receptor complexes (Aqvist et al., 2002; Carlsson et al., 2008; Kraszewski et al., 2010). The $\gamma$ constant was set as ~ -7.0 kcal/mol in order to reproduce binding affinities previously reported for the isoflurane/NaChBac system (see below). Although important for proper estimation of absolute binding free energies within the LIE framework, the choice of $\alpha$, $\beta$ and $\gamma$ must be, however, less critical for comparative analysis of relative binding energies of the ligand against multiple sites, which is the underlying scenario of the present study. In Table B, standard deviation were estimated by taking into account at least two independent estimates of sevoflurane affinities per binding site. The equilibrium constant of sevoflurane binding per site was computed from eq. [1] by assuming quadratic fluctuations of the ligand in the bound state

$K \approx\frac{1}{8\pi^{2}}\Delta V\Delta\omega e^{-\beta\Delta G_{bind}}$ [4]

where, $\Delta\omega$ and $\Delta V$ denote respectively the orientational and translational freedom of the ligand in the bound complex (Luo and Sharp, 2002; Swanson et al., 2004; Woo and Roux, 2005). Eq. [4] assumes that the volumes of the configuration space related to internal degrees of freedom of the ligand and protein change negligibly upon association. By using the quasiharmonic approximation, $\Delta\omega$ and $\Delta V$ were respectively estimated here as typical Euler angle fluctuations and center-of-mass fluctuations of the ligand in the binding site (Swanson et al., 2004). The MD-generated ensemble was considered for that purpose. Note that, the equilibrium binding constant in eq. [4] has units of inverse density number $Å^{3}$; multiplication by ${6.02 x 10}^{-4}$ converts to concentration units $M^{-1}$. From eq. [4], a per site absolute binding energy was defined as

$\Delta G_{bind}^{0}= -\beta^{-1}ln(K C^{0})$ [5]

in which $C^{0}$ is a standard state concentration of $1 M$.

**Molecular Dynamics.** All MD simulations were carried out using the program NAMD 2.9 (Philips et al., 2005). Langevin dynamics and Langevin piston methods were applied to keep the temperature (300 K) and the pressure (1 atm) of the system fixed. The equations of motion were integrated using a multiple time-step algorithm (Izaguirre et al., 1999). Short- and long-range forces were calculated every 1 and 2 time-steps respectively, with a time step of 2.0 fs. Also, periodic-boundary conditions were employed**.** Chemical bonds between hydrogen and heavy atoms were constrained to their equilibrium value. Long-range electrostatic forces were taken into account using the Particle Mesh Ewald (PME) approach (Darden et al., 2003). The CHARMM36 force field (Huang and MacKerell, 2013) was applied and water molecules were described by the TIP3P model (Jorgensen et al., 1983). Charmm-based parameters for sevoflurane were obtained from the molecular model of the ligand devised by Barber et al. (Barber et al., 2014). All the protein charged amino acids were simulated in their full-ionized state (pH=7.0). A force constant of ~10.0 kcal/mol/Å^2^ was considered in LIE related simulations of the ligand-bound state. Simulations were performed on local HPC facility at LBTC.

**Validation of Modeling Approach**

The accuracy of the docking/LIE approach in describing the interaction mode of anesthetics and ion channels was confronted to recent studies by Klein and coworkers on the isoflurane/NaChBac system (Raju et al., 2013). In detail, flooding MD simulations were applied to identify isoflurane occupancy sites on NaChBac followed by quantification of binding affinities via the free-energy perturbation (FEP) method. The study showed a higher occupancy of isoflurane at four independent sites, named by the authors as extracellular, S4-S5 linker, pore and fenestrations. Absolute binding free energies of -4.2 ± 0.8 and -3.7 ± 0.4 kcal/mol were respectively reported for the interaction of the ligand at the extracellular and linker sites. In order to reproduce these results following the docking/LIE approach, the (resting/closed), NaChBac structure as modeled by Barber et al. (Barber et al., 2012) was first MD simulated in the membrane and subjected to isoflurane docking calculations. This approach, which is independent from that used in the flooding simulations allowed us to properly identify among others ( Figure F), the isoflurane binding sites described by Klein and coworkers (Raju et al., 2013). The LIE method allowed us to calculate binding affinities for each site, according to eq. [2], following calibration of the parameters in eq. [1]. After proper calculations of translation and rotational freedom of the bound ligand from the MD simulations and applying formulation given by eq. [5], LIE-based calculations yielded absolute binding energies that are in good agreement with those obtained by means of FEP calculations (Table C). Figure G presents the time series of the van der Waals $V^{vdW}$ and electrostatic $V^{elect}$ interaction energies of isoflurane in its receptor-bound and solution-free states. Taken together, these findings justify the docking/LIE as a reliable and less cumbersome approach for the investigation of anesthetic binding to ion channels.

**Supplemental References**

Aqvist J, Medina C. and Samuelsson JE (1994) A new method for predicting binding affinity in computer-aided drug design. *Protein Eng.* **7:**385–391.

Aqvist J, Luzhkov VB and Brandsdal BO (2002) Ligand binding affinities from MD simulations. *Acc. Chem. Res.* **35:**358–365.

Barber AF, Carnevale V, Raju SG, Amaral C, Treptow W and Klein ML(2012) Hinge-bending motions in the pore domain of a bacterial voltage-gated sodium channel. *Biochim. Biophys. Acta* **1818:**2120–2125.

Barber AF, Carnevale V, Klein ML, Eckenhoff RG and Covarrubias M (2014) Modulation of a voltage-gated Na^+^ channel by sevoflurane involves multiple sites and distinct mechanisms. *Proc. Natl. Acad. Sci.* **111:**6726–6731.

Carlsson J, Boukharta L and Aqvist J (2008) Combining docking, molecular dynamics and the linear interaction energy method to predict binding modes and affinities for non-nucleoside inhibitors to HIV-1 reverse transcriptase. *J. Med. Chem.* **51:**2648–2656.

Darden T, York D and Pedersen L (1993) Particle mesh Ewald: An Nlog(N) method for Ewald sums in large systems. *J. Chem. Phys.* **98:**10089–10092.

Huang J and MacKerell AD (2013) CHARMM36 all-atom additive protein force field: Validation based on comparison to NMR data. *J. Comput. Chem.* **34:**2135–2145.

Izaguirre JA, Reich S and Skeel RD (1999) Longer time steps for molecular dynamics. *J. Chem. Phys.* **110:**9853–9864.

Jorgensen WL, Chandrasekhar J, Madura JD, Impey RW and Klein ML (1983) Comparison of simple potential functions for simulating liquid water. *J. Chem. Phys.* **79:**926–935.

Luo H and Sharp K. On the Calculation of Absolute Macromolecular Binding Free Energies. Proc Natl Acad Sci USA, **99:**10399-10404, 2002.

Kraszewski S, Tarek M, Treptow W and Ramseyer C (2010) Affinity of C60 neat fullerenes with membrane proteins: a computational study on potassium channels. *ACS Nano* **4:**4158–4164.

[Phillips JC](http://www.ncbi.nlm.nih.gov/pubmed/?term=Phillips%20JC%5BAuthor%5D&cauthor=true&cauthor_uid=16222654), [Braun R](http://www.ncbi.nlm.nih.gov/pubmed/?term=Braun%20R%5BAuthor%5D&cauthor=true&cauthor_uid=16222654), [Wang W](http://www.ncbi.nlm.nih.gov/pubmed/?term=Wang%20W%5BAuthor%5D&cauthor=true&cauthor_uid=16222654), [Gumbart J](http://www.ncbi.nlm.nih.gov/pubmed/?term=Gumbart%20J%5BAuthor%5D&cauthor=true&cauthor_uid=16222654), [Tajkhorshid E](http://www.ncbi.nlm.nih.gov/pubmed/?term=Tajkhorshid%20E%5BAuthor%5D&cauthor=true&cauthor_uid=16222654), [Villa E](http://www.ncbi.nlm.nih.gov/pubmed/?term=Villa%20E%5BAuthor%5D&cauthor=true&cauthor_uid=16222654), [Chipot C](http://www.ncbi.nlm.nih.gov/pubmed/?term=Chipot%20C%5BAuthor%5D&cauthor=true&cauthor_uid=16222654), [Skeel RD](http://www.ncbi.nlm.nih.gov/pubmed/?term=Skeel%20RD%5BAuthor%5D&cauthor=true&cauthor_uid=16222654), [Kalé L](http://www.ncbi.nlm.nih.gov/pubmed/?term=Kal%C3%A9%20L%5BAuthor%5D&cauthor=true&cauthor_uid=16222654) and [Schulten K](http://www.ncbi.nlm.nih.gov/pubmed/?term=Schulten%20K%5BAuthor%5D&cauthor=true&cauthor_uid=16222654) (2005) Scalable molecular dynamics with NAMD. *J. Comput. Chem****.* 26:**1781–1802.

Raju SG, Barber AF, LeBard DN, Klein ML and Carnevale V (2013) Exploring Volatie General Anesthetic Binding to a Closed Membrane-Bound Bacterial Voltage-Gated Sodium Channel via Computation. *Plos Comput Biol* **9:**1003090.

Swanson JM, Henchman RH, McCammon JA. Revisiting Free Energy Calculations: A Theoretical Connection to MM/PBSA and Direct Calculation of the Association Free Energy. Biophys J, **86:**67-74, 2004.

Trott O and Olson AJ (2010) AutoDock Vina: Improving the speed and accuracy of docking with a new scoring function, efficient optimization, and multithreading. *J. Comput. Chem.* **31:**455–461.

Woo HJ and Roux B. Calculation of absolute protein-ligand binding free energy from computer simulations. Proc Natl Acad Sci USA, **1102:**6825-6830, 2005.

**Supplemental Figures**

**

**

**Figure A. *G*-*V* relations of Kv1.2 S4-S5 linker mutants.**

(A) Families of whole-oocyte currents of Kv1.2 S4-S5 linker mutants including L321F, K322R, M325A and R326K. The scale bars indicate 100 ms and 0.5 µA. (B) *G*-*V* relations of Kv1.2 and the four mutants. The solid lines are the best fits to the Boltzmann equation.





**Figure B. Positive modulation of Kv1.2-FRAKT by sevoflurane is T1 domain-independent.**

(A) Families of whole-oocyte ΔT1-Kv1.2-FRAKT currents in the absence (top) and presence of 1 mM sevoflurane (bottom). The scale bars indicate 100 ms and 1 µA. (B) *G*-*V* relations of ΔT1-Kv1.2-FRAKT in the absence (open) and presence of 1 mM sevoflurane (filled). The solid lines are the best fits to double Boltzmann equation. For comparison to full-length channel, the grey and red lines are the best fits to a double Boltzmann equation for Kv1.2-FRAKT in the absence (grey) and presence of 1 mM sevoflurane (red). These results are replotted from Fig. 6C.

|...S4-S5 linker...|........S5.........|

rKv1.1 VFRIFKLSRHSKGLQILG-QTLKASMRELGLLIFFLFIGVILFSSAVYFA 344

rKv1.2 VFRIFKLSRHSKGLQILG-QTLKASMRELGLLIFFLFIGVILFSSAVYFA 346

rKv2.1 ILRILKLARHSTGLQSLG-FTLRRSYNELGLLILFLAMGIMIFSSLVFFA 344

rKv3.1 ILRIFKLTRHFVGLRVLG-HTLRASTNEFLLLIIFLALGVLIFATMIYYA 366

dShaw IMRLFKLTRHSSGLKILI-QTFRASAKELTLLVFFLVLGIVIFASLVYYA 347

rKv4.1 VFRIFKFSRHSQGLRILG-YTLKSCASELGFLLFSLTMAIIIFATVMFYA 344

rKv5.1 IARIFKLARHSSGLQTLT-YALKRSFKELGLLLMYLAVGIFVFSALGYTM 353

rKv6.1 ILYVMRLARHSLGLQTLG-LTARRCTREFGLLLLFLCVAIALFAPLLYVI 395

rKv7.1 ILRMLHVDRQGGTWRLLG-SVVFIHRQELITTLYIGFLGLIFSSYFVYLA 281

rkV8.1 ALRMLKLGRHSTGLRSLG-MTITQCYEEVGLLLLFLSVGISIFSTIEYFA 366

rKv9.1 IFRVLKLARHSTGLRSLG-ATLKHSYREVGILLLYLAVGVSVFSGVAYTA 365

rKv10.1 LLRLGRVARKLDHYIEYGAAVLVLLVCVFGLAAHWMACIWYSIGDYEIFD 380

rKv11.1 LLRLVRVARKLDRYSEYGAAVLFLLMCTFALIAHWLACIWYAIGNMEQPH 576

rKv12.1 LLRLLRLLQKLDRYSQHSTIVLTLLMSMFALLAHWMACIWYVIGKMER-E 383

KvAP LLRFLRILLIISRGSKFLSAIADAADKIRFYHLFGAVMLTVLYGAFAIYI 180

**Figure C. Sequence alignment of the S4-S5 linkers from selected Kv channels.**

Kv1.2-G329 and amino acids at equivalent position are highlighted in red.


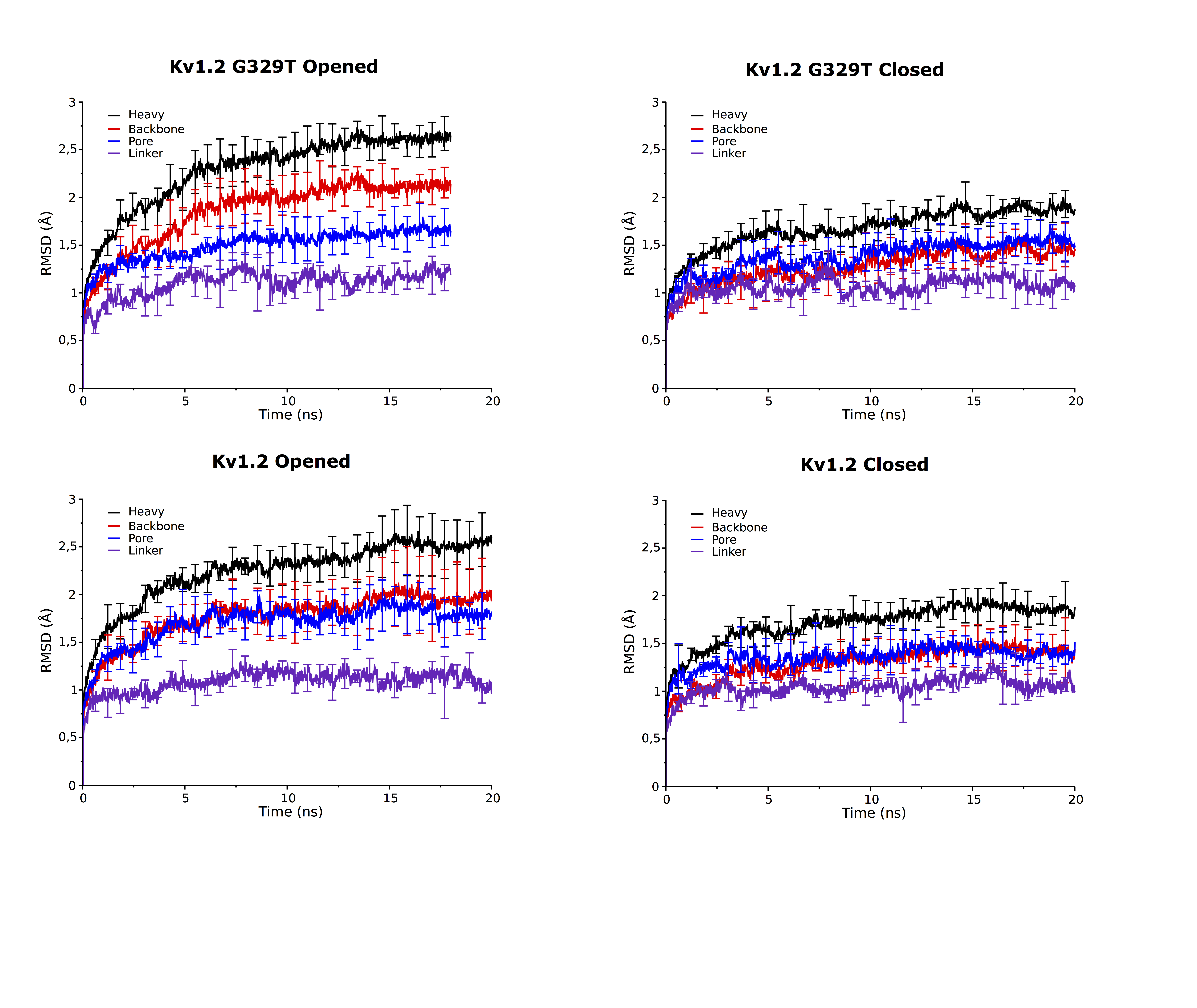


**Figure D. Root Mean Square Deviation (RMSD) plots for Kv1.2 and Kv1.2-G329T inserted in a POPC bilayer in relation to the starting structure.** Different plots correspond to all atoms in the channel structure excluding hydrogen atoms (black), backbone atoms (red), pore atoms (blue) and S4-S5 linker atoms (purple).


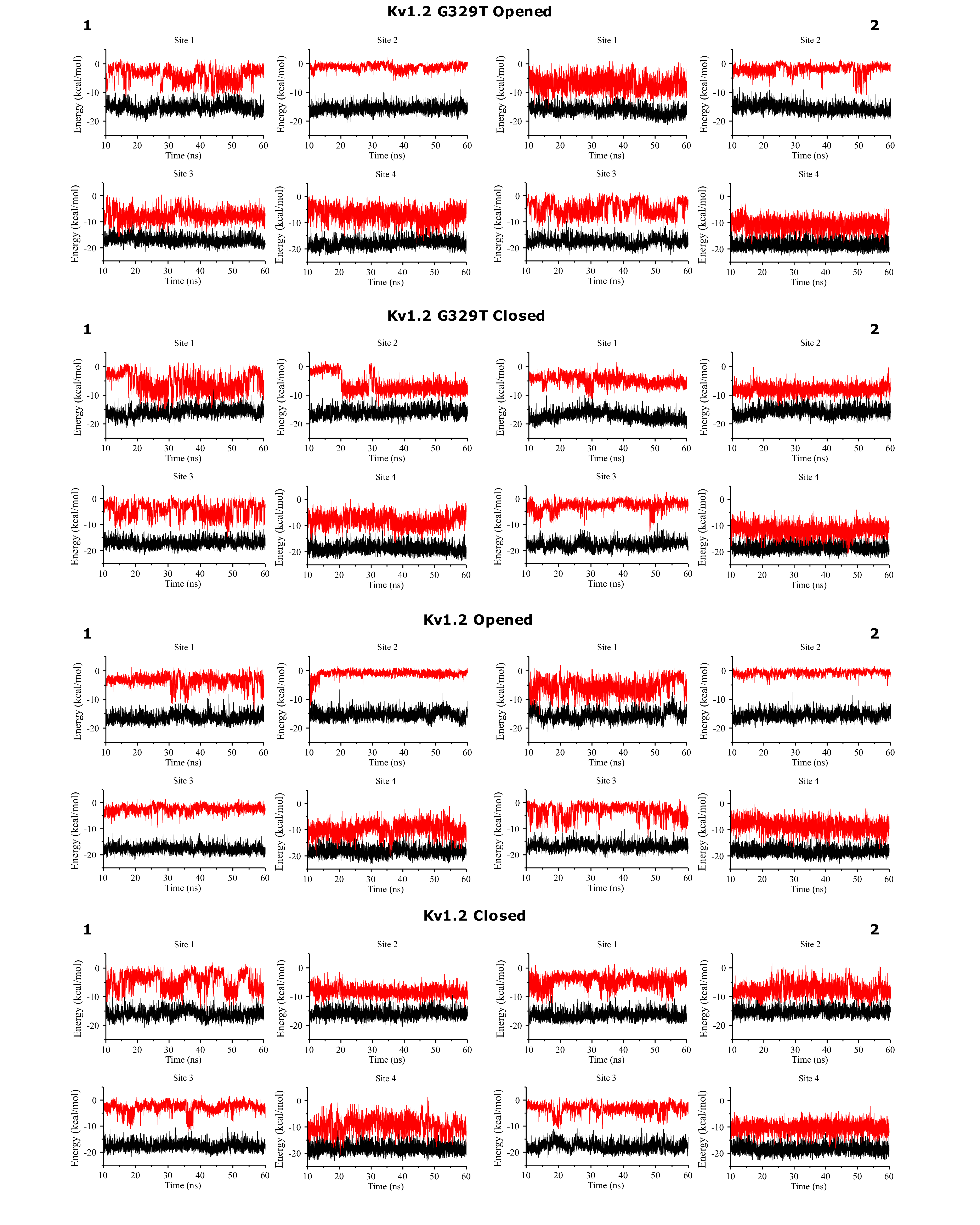


**Figure E. Non-bonded interaction energy for each sevoflurane molecule according to site (1 to 4) and channel (Kv1.2 and Kv1.2-G329T), and dependent on state (open or closed).** Van der Waals (black) and eletrostatic (red) potentials are plotted against time. For every site, the energy time series are averages over all channel subunits.


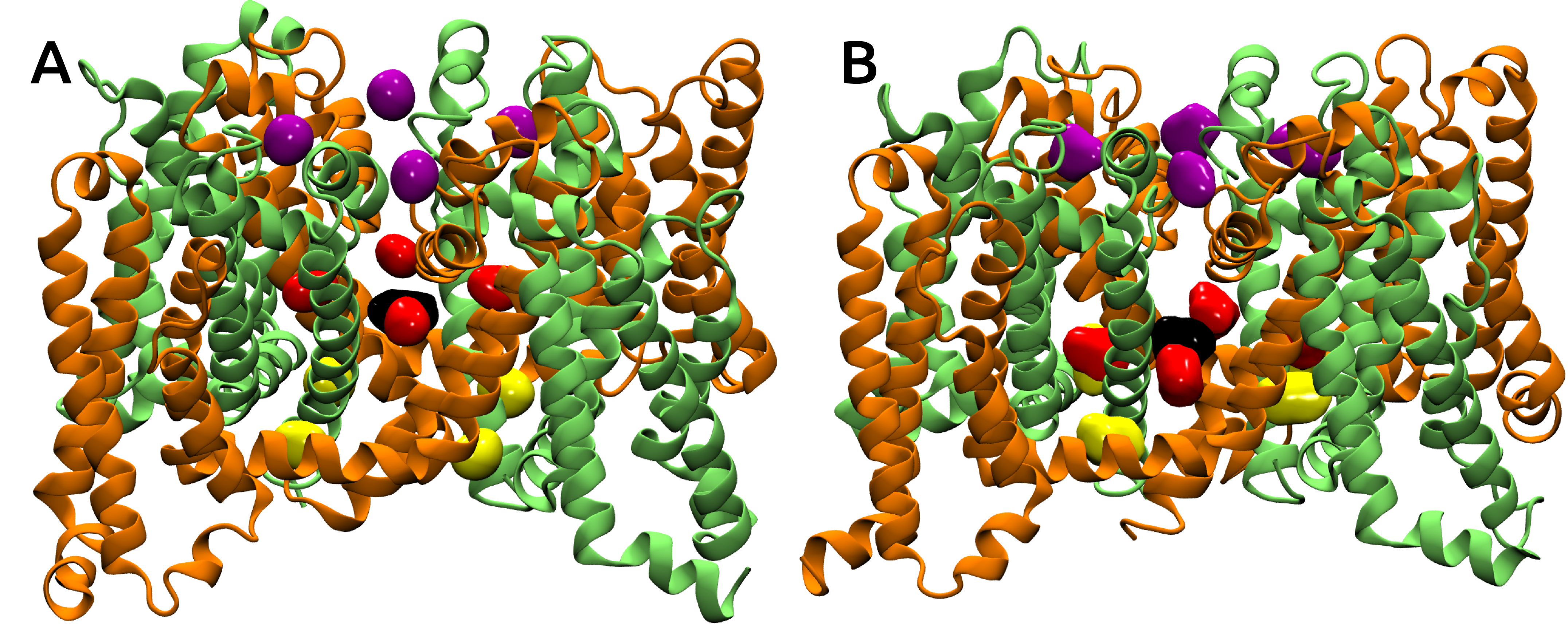


**Figure F. (A) Regions with highest isoflurane occupancy during the flooding simulation.** The extracellular site is in purple, the fenestrations site in red, the pore site in black and the linker site in yellow. **(B) Binding sites of isoflurane identified by docking on NachBac.** Each site color is the same as described in A.


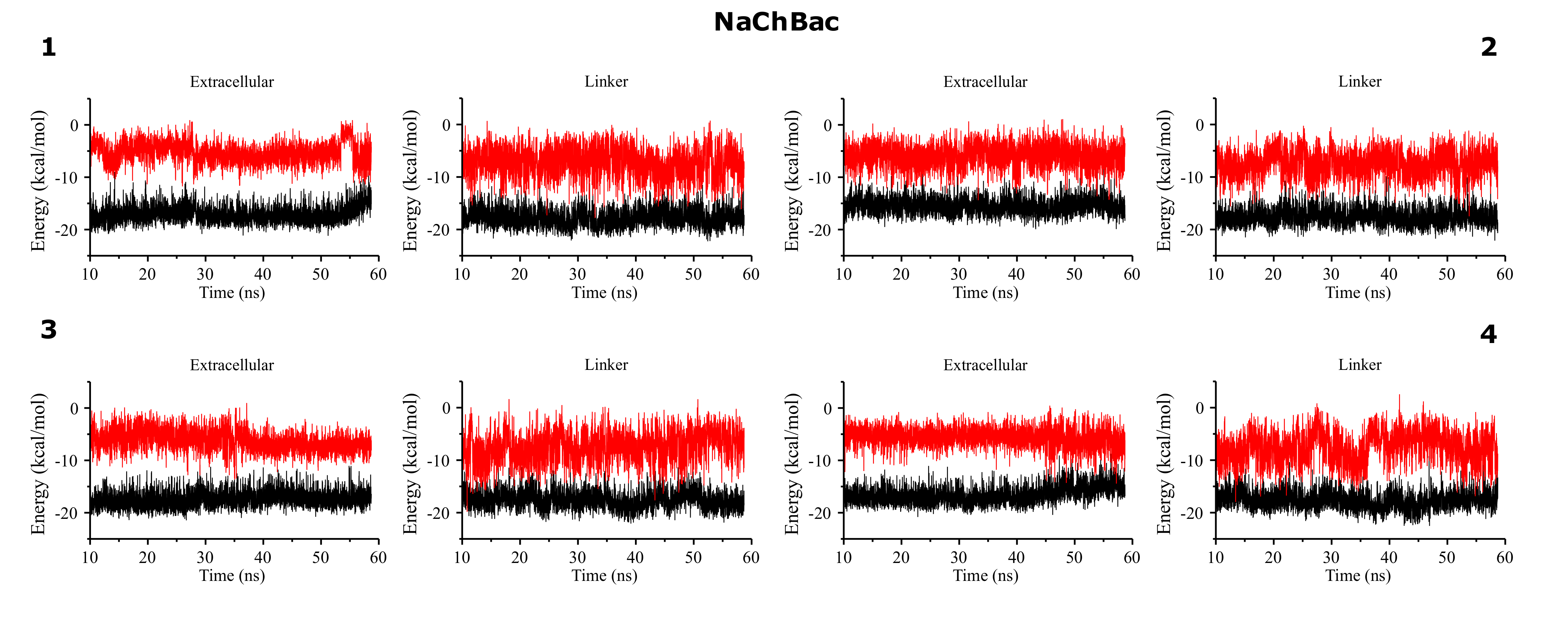


**Figure G. Surrounding interaction energy for each isoflurane molecule (1, 2, 3 and 4) according to site (Extracellular and Linker) and dependent on state (open or closed).** Van der Waals (black) and Eletrostatic (red) potentials are plotted against time.

**Tables**

**Table A. *G*-*V* parameters of selected Kv channels in the absence and presence of 1 mM sevoflurane**

**P*<0.05, ***P*<0.01, ****P*<0.001 compared to control by using paired Student *t*-test.

§ The statistical significance of *G*_max_ changes was evaluated from the raw values before normalizing (main text, Figs. 2 and 7).

|  | *V*_1/2,1_  (mV) | *z*_1_  (e_0_) | *G*_max,1_^§^ | *V*_1/2,2_  (mV) | *z*_2_  (e_0_) | *G*_max,2_^§^ | *V*_med_  (mV) |
| --- | --- | --- | --- | --- | --- | --- | --- |
| **Kv1.2**  (n=6) |  |  |  |  |  |  |  |
| Control | -15.4±1.2 | 2.96±0.11 | 1 |  |  |  |  |
| Sevoflurane | -19.5±0.8** | 2.89±0.14 | 1.13±0.03 |  |  |  |  |
| **ΔT1-Kv1.2**  (n=6) |  |  |  |  |  |  |  |
| Control | -21.9±3.2 | 3.85±0.14 | 1 |  |  |  |  |
| Sevoflurane | -25.8±3.4* | 4.00±0.21 | 1.10±0.02 |  |  |  |  |
| **Kv1.2 FRAKT**  (n=6) |  |  |  |  |  |  |  |
| Control | -18.1±1.9 | 2.75±0.10 | 0.37±0.01 | 60.4±1.3 | 0.85±0.03 | 0.63±0.01 | 21.9±2.3 |
| Sevoflurane | -10.9±2.5*** | 3.16±0.04* | 0.99±0.04 | 52.3±4.3 | 1.03±0.03* | 1.07±0.05 | 6.6±3.5** |
| **ΔT1-Kv1.2 FRAKT**  (n=5) |  |  |  |  |  |  |  |
| Control | -17.0±2.3 | 2.63±0.21 | 0.33±0.02 | 55.7±3.8 | 1.0±0.1 | 0.9967±0.05 | 28.5±4.3 |
| Sevoflurane | -12.9±2.1 | 3.04±0.12 | 0.84±0.07 | 47.3±6.3 | 1.1±0.1 | 1.08±0.06 | 9.2±2.2* |
| **Kv1.2 G329T**  (n=4) |  |  |  |  |  |  |  |
| Control | -5.2±1.3 | 4.8±0.1 | 0.30±0.01 | 44.0±1.5 | 1.5±0.04 | 0.70±0.01 | 28.3±2.4 |
| Sevoflurane | -6.4±1.2 | 5.0±0.3 | 0.77±0.03 | 33.0±1.9* | 1.5±0.1 | 1.09±0.10 | 8.4±1.2** |
| **K-Shaw2**  (n=4) |  |  |  |  |  |  |  |
| Control | 36.4±3.3 | 1.17±0.06 | 1 |  |  |  |  |
| Sevoflurane | 10.2±7.2* | 1.16±0.05 | 1.60±0.11 |  |  |  |  |
| **K-Shaw2 T330G**  (n=6) |  |  |  |  |  |  |  |
| Control | 30.6±9.0 | 0.93±0.04 | 1 |  |  |  |  |
| Sevoflurane | 24.0±3.3 | 1.05±0.06 | 1.41±0.10 |  |  |  |  |
| **ΔT1-K-Shaw2**  (n=6) |  |  |  |  |  |  |  |
| Control | -11.1±4.2 | 1.29±0.09 | 1 |  |  |  |  |
| Sevoflurane | -15.4±5.5 | 1.24±0.12 | 1.01±0.01 |  |  |  |  |

**Table B. Computed values of binding energies of sevoflurane against Kv1.2 and Kv1.2 G329T.**

Molecular Dynamics averages of the van de Waals ($\left\langle V^{vdW} \right\rangle$)and Eletrostatic ($\left\langle V^{elect} \right\rangle$) potentials, Binding constants ($K_{b}$), Orientational ($\Delta\Omega$) and Translational ($\Delta V$) freedom of the ligand, Binding Free Energies ($\Delta G_{bind}^{calc}$) and Absolute Binding Free Energies ($\Delta G_{bind}^{0}$) obtained from the LIE method .

| **Site** | **Isoform** | $\left\langle V^{el} \right\rangle$**^*^** | $\left\langle V^{vdw} \right\rangle$**^*^** | $\Delta G_{bind}^{calc}$^*^ | $K_{B}$^#^ | $\Delta V$ ^@^ | $\Delta\Omega$ ^##^ | $\Delta G_{bind}^{0}$ |
| --- | --- | --- | --- | --- | --- | --- | --- | --- |
| **Site 1** | **Kv1.2 G329T O** | -6.42 ± 0.48 | -15.52 ± 0.62 | -6.84 ± 0.97 | 0.2228 | 16.36 | 16.06 | -3.22 |
|  | **Kv1.2 G329T C** | -5.19 ± 0.60 | -16.65 ± 0.54 | -6.58 ± 0.98 | 0.1196 | 14.17 | 16.27 | -2.85 |
|  | **Kv1.2 O** | -4.32 ± 0.86 | -15.44 ± 0.90 | -6.12 ± 1.00 | 0.0490 | 8.74 | 25.50 | -2.32 |
|  | **Kv1.2 C** | -4.72 ± 0.01 | -16.58 ± 0.44 | -6.43 ± 0.96 | 0.1102 | 12.82 | 21.98 | -2.80 |
| **Site 2** | **Kv1.2 G329T O** | -1.94 ± 0.68 | -15.87 ± 0.26 | -5.37 ± 0.98 | 0.0160 | 8.55 | 30.02 | -1.65 |
|  | **Kv1.2 G329T C** | -7.16 ± 0.95 | -15.91 ± 0.01 | -7.16 ± 1.00 | 0.1108 | 3.58 | 26.47 | -2.81 |
|  | **Kv1.2 O** | -1.06 ± 0.32 | -15.22 ± 0.12 | -4.97 ± 0.96 | 0.0182 | 19.20 | 26.90 | -1.73 |
|  | **Kv1.2 C** | -7.95 ± 0.49 | -15.67 ± 0.36 | -7.39 ± 0.97 | 0.1329 | 2.64 | 23.24 | -2.91 |
| **Site 3** | **Kv1.2 G329T O** | -6.25 ± 1.29 | -17.33 ± 0.28 | -7.07 ± 1.05 | 0.1838 | 8.66 | 20.25 | -3.11 |
|  | **Kv1.2 G329T C** | -3.55 ± 0.79 | -17.22 ± 0.32 | -6.14 ± 0.99 | 0.0534 | 8.18 | 29.24 | -2.37 |
|  | **Kv1.2 O** | -2.96 ± 0.37 | -16.87 ± 0.31 | -5.88 ± 0.96 | 0.0184 | 3.90 | 31.41 | -1.73 |
|  | **Kv1.2 C** | -3.24 ± 0.23 | -17.68 ± 0.10 | -6.11 ± 0.96 | 0.0579 | 6.62 | 39.13 | -2.42 |
| **Site 4** | **Kv1.2 G329T O** | -9.42 ± 1.70 | -18.01 ± 0.35 | -8.12 ± 1.11 | 0.4081 | 7.62 | 12.60 | -3.58 |
|  | **Kv1.2 G329T C** | -9.42 ± 2.15 | -18.59 ± 0.05 | -8.35 ± 1.20 | 0.3721 | 4.33 | 9.66 | -3.53 |
|  | **Kv1.2 O** | -8.10 ± 0.61 | -18.33 ± 0.03 | -8.15 ± 0.99 | 0.3656 | 3.38 | 16.93 | -3.52 |
|  | **Kv1.2 C** | -8.85 ± 0.82 | -18.47 ± 0.39 | -8.14 ± 1.00 | 0.2955 | 3.11 | 14.51 | -3.39 |

For this calculation, sevoflurane potentials in the water reference were $\left\langle V^{vdw} \right\rangle=-11.39\pm1.49$ and $\left\langle V^{el} \right\rangle=-8.53\pm2.72$ and empirical values adopted were $\alpha=0.18$, $\beta=0.34$ and $\gamma=-6.90{kcal}/{mol}$. * kcal/mol; ^#^mM^-1^; ^##^${rad}^{3};$ ^@^ $Å^{3}$

**Table C. Computed values of binding energies of isoflurane against NaChBac.** Molecular Dynamics averages of the van der Waals ($\left\langle V^{vdW} \right\rangle$) and Eletrostatic ($\left\langle V^{elect} \right\rangle$) potentials, Orientational (∆Ω) and Translational (∆V) freedom of the ligand, Binding Free Energies ($\Delta G_{bind}^{calc}$) and Absolute Binding Free Energies ($\Delta G_{bind}^{0}$) obtained from the LIE method.

| **Site** | **Isoform** | $\left\langle V^{el} \right\rangle$***** | $\left\langle V^{vdw} \right\rangle$***** | $\Delta G_{bind}^{calc}$^*^ | $K_{B}$^#^ | $\Delta V$ ^@^ | $\Delta\Omega$ ^##^ | $\Delta G_{bind}^{0}$***** |
| --- | --- | --- | --- | --- | --- | --- | --- | --- |
| **Extracellular** | **NaChBac** | -5.44 ± 0.41 | -16.67 ± 0.73 | -7.48 ± 0.84 | 0.4443 | 16.34 | 13.44 | -3.63 |
| **Linker** | **NaChBac** | -7.50 ± 0.05 | -17.64 ± 0.08 | -8.34 ± 0.82 | 2.2217 | 8.69 | 28.12 | -4.59 |

For this calculation, isoflurane potentials in the water reference were $\left\langle V^{vdw} \right\rangle=-11.60\pm1.43$ and $\left\langle V^{el} \right\rangle=-6.10\pm2.30$ and empirical values adopted were $\alpha=0.18$, $\beta=0.34$ and $\gamma=-6.90{kcal}/{mol}$. *kcal/mol.
